# Recurrent polymorphic mating type variation in Madagascan *Bulbophyllum* species (Orchidaceae) exemplifies a high incidence of auto-pollination in tropical orchids

**DOI:** 10.1111/boj.12168

**Published:** 2014-05-20

**Authors:** Alexander Gamisch, Gunter A Fischer, Hans Peter Comes

**Affiliations:** 1 Department of Organismic Biology, University of SalzburgA-5020, Salzburg, Austria; 2 Kadoorie Farm and Botanic Garden CorporationLam Kam Road, Tai Po, N.T., Hong Kong SAR

**Keywords:** autogamy, breeding system, evolution, floral morphology, light microscopy, polymorphism, scanning electron microscopy

## Abstract

The transition from outcrossing to self-fertilization is one of the most common evolutionary changes in angiosperms. The orchid family exemplifies this evolutionary trend but, because of a general lack of large-scale surveys on auto-pollination in orchid taxa, the incidence and modes of auto-pollination among (sub)tropical orchids remain poorly known. In the present study, we assessed the frequency and mode of auto-pollination within and among species of a largely monophyletic group of Madagascan *Bulbophyllum*. The capacity for autonomous fruit set was investigated by bagging experiments in the greenhouse and the field, complemented with detailed floral micromorphological studies of the gynostemium. Our survey comprises 393 accessions, representing at least 78 species, and thus approximately 37% of the species diversity of the genus in the Madagascan region. Our studies revealed that mating type is directly related to gynostemium structure, most often involving the presence or absence of a physical barrier termed ‘rostellum’. As a novel and unexpected finding, we identified eight species of a single lineage of Madagascan *Bulbophyllum* (termed ‘clade C’), in which auto-pollinating morphs (selfers), either lacking a rostellum or (rarely) possessing a stigmatic rostellum, co-exist with their pollinator-dependent conspecifics (outcrossers). We hypothesize that auto-pollination via rostellum abortion has a simple genetic basis, and probably evolved rapidly and recurrently by subtle changes in the timing of rostellum development (heterochrony). Thus, species of clade C may have an intrinsic genetic and developmental lability toward auto-pollination, allowing rapid evolutionary response under environmental, perhaps human-disturbed conditions favouring reproductive assurance. Overall, these findings should stimulate further research on the incidence, evolution, and maintenance of mating type variation in tropical orchids, as well as how they adapt(ed) to changing environmental conditions.

## Introduction

The complex flower structures and pollination systems of orchids have offered some of the most spectacular and best-documented examples of the intense selective pressures for outcrossing in flowering plants to avoid inbreeding (Catling, 1990). Darwin (1862, 1877) was the first to draw attention to this significant evolutionary topic, although he was also well aware of approximately 23 orchid species capable of autonomous within-flower self-pollination (autogamy) without the aid of a vector (auto-pollination *sensu* Catling, 1990) and numerous other species have subsequently been added to this initially short list (Ridley, 1888; Reiche, 1910; Kirchner, 1922; Catling, 1990; Liu *et al*., 2006; Zhou *et al*., 2012). Darwin (1862, 1877) was also the first to suggest that some orchid species have become secondarily modified for auto-pollination in habitats that prohibit pollen dispersal, or where conspecific mates are scarce (e.g. after a founder event). There is now abundant support for this ‘reproductive assurance’ hypothesis (Baker, 1955; Jain, 1976; Charlesworth, 2006; but see also Busch, Joly & Schoen, 2011), which also concurs with the tendency of auto-pollinating orchids to be relatively frequent in geographically isolated and/or pollinator-scarce environments, such as higher latitudes/elevations, coastal areas, islands, and distributional range limits (Hagerup, 1952; Bates, 1978; Catling, 1990; Jacquemyn *et al*., 2005; Zhou *et al*., 2012). In addition, theory predicts that a selfing mutation will rapidly spread in a randomly mating population as a result of the increased transmission of gametes to the next generation (Fisher, 1941; Jain, 1976; Charlesworth, 2006; Cheptou, 2012). However, strict empirical tests of this ‘automatic selection advantage of selfing’ are hard to come by, and have rarely been conducted in orchid systems (Ortiz-Barney & Ackerman, 1999).

Auto-pollination has been reported in almost every tribe and subtribe of Orchidaceae, and most, if not all, auto-pollinating orchids have close pollinator-dependent relatives (Arroyo, 1973; Catling, 1990). Moreover, both auto-pollinating (‘selfing’) and pollinator-depending (‘outcrossing’) individuals, populations and races have been reported within orchid species [e.g. *Bulbophyllum* Thouars: Schlechter, 1914; Kirchner, 1922; Smith, 1928; *Spiranthes sinensis* (Pers.) Ames: Bates, 1978; *Cephalanthera* Rich.: Scacchi, De Angelis & Corbo, 1991; *Epipactis* Zinn: Ehlers & Pedersen, 2000; *Angraecum* Bory: Jacquemyn *et al*., 2005; *Eulophia* R.Br.: Peter & Johnson, 2009]. Auto-pollination in orchids is generally considered to have evolved independently on many occasions, most probably from ancestrally outcrossing conditions (Catling, 1990; see also Stebbins, 1957), although firm (e.g. phylogenetic) evidence in support of this remains scarce (Hapeman & Inoue, 1997).

Approximately 31% of orchid species in which the pollination system has been investigated are capable of auto-pollination (Peter, 2009; Peter & Johnson, 2009; Zhou *et al*., 2012), suggesting that auto-pollination is indeed common in Orchidaceae (van der Cingel, 1995, 2001). However, this estimate is based on data representing only approximately 8% of all known orchid species (> 25 000; Dressler, 1981), and approximately two-thirds of the reported auto-pollinators are found among the 25% of terrestrial orchids, mainly from northern temperate and boreal regions (Catling, 1990). Hence, the commonly held notion that auto-pollinating orchids predominate in relatively colder climates and/or at higher latitudes (Hagerup, 1952; Catling, 1990) must be viewed with scepticism, given the relative lack of information on the pollination system of (mainly epiphytic) orchid genera from tropical regions (approximately 70% of the total; Dressler, 1981; Gravendeel *et al*., 2004). Nonetheless, Catling (1990: 145) speculated that auto-pollination in orchids may be much less of a selective advantage and, consequently, not as frequent, in (sub)tropical regions, ‘where environments have been more stable, except with regard to the recent past’ (i.e. as a result of human disturbance, such as clearing and selective logging; Breed *et al*., 2012). However, there is little evidence yet from the literature that the evolution of auto-pollination is constrained in tropical orchids (Jacquemyn *et al*., 2005), although the circumstances under which it may confer an advantage remain to be adequately explored.

Orchids usually avoid selfing by separating the anther and stigma by a rostellum, which is generally derived from the distal portion of the median stigma lobe (Kurzweil & Kocyan, 2002; Kurzweil, Weston & Perkins, 2005; Luo, Zhu & Kurzweil, 2005; Efimov, 2011). In most auto-pollinating orchids, the rostellum either does not develop or, more rarely, develops incompletely or secondarily disintegrates, allowing the pollinia and stigma to come into contact (Catling, 1990). Less frequent modes of auto-pollination involve (1) the over-secretion of the stigma (Catling, 1990); (2) the movement of the perianth, anther(s), or pollinia (Catling, 1990; Liu *et al*., 2006); (3) the falling of friable pollinia onto the stigmatic surface(s) (Hagerup, 1952); and (4) the stigmatic functioning of the rostellum (Williamson, 1984; Gamisch *et al*., 2013). Moreover, a new mode of auto-pollination has recently been discovered in a slipper orchid from south-western China (Chen *et al*., 2012), involving the sliding of a liquefied anther onto the stigmatic surface. However, the incidence and modes of auto-pollination among (sub)tropical orchids are still poorly documented and little understood.

**Figure 1:**
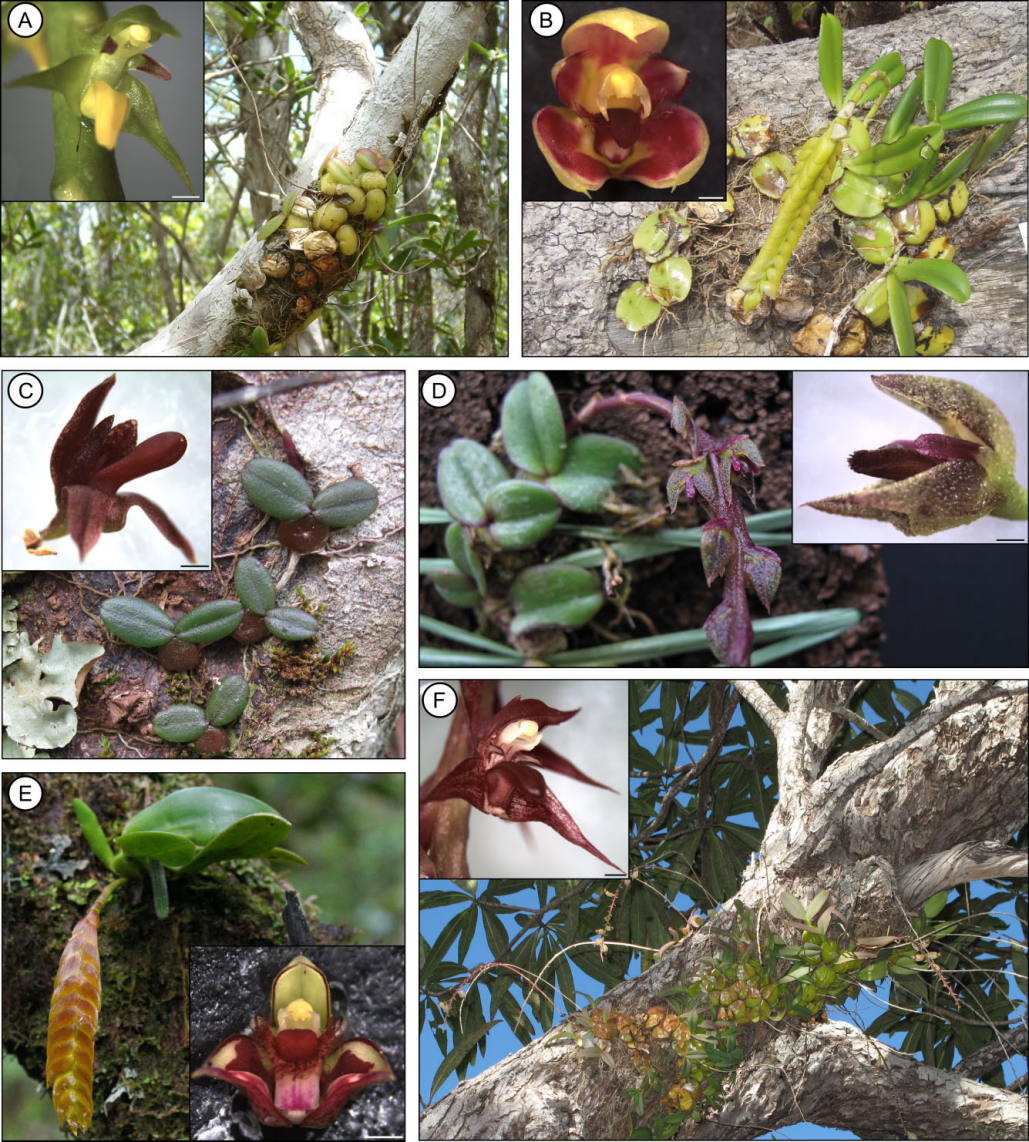
Habit and flower close-ups of six Madagascan *Bulbophyllum* clade C species. A, *Bulbophyllum pervillei*. B, *Bulbophyllum cirrhoglossum*. C, *Bulbophyllum* sp. A. D, *Bulbophyllum ruginosum*. E, *Bulbophyllum occultum*. F, *Bulbophyllum histrionicum*. Scale bar = 1 mm. All habit photographs by G. A. Fischer and A. Sieder (only D); all flower close ups by A. Gamisch.

*Bulbophyllum* (Epidendroideae) is one of the most species-rich orchid genera (approximately 2400 species; Sieder, Rainer & Kiehn, 2007) and offers compelling opportunities for deriving principles of mating system shifts in tropical orchids. Most species of this largely self-compatible genus occur as epiphytes (Fig. 1) or, more rarely, litho- and rheophytes, in the (sub)tropical rain and cloud forests of South-East Asia, Africa, South America, and especially richly in the montane forests of New Guinea and Madagascar (Fischer *et al*., 2007; Vermeulen & Tsukaya, 2011). *Bulbophyllum* flowers are usually adapted to cross-pollination mediated by flies (Bartareau, 1994; Tan, Tan & Nishida, 2006; Liu *et al*., 2010; Humeau *et al*., 2011) or, more rarely, wasps and bees (van der Cingel, 2001; Chen & Gao, 2011). Despite varying reports on the reproductive biology and micromorphology of *Bulbophyllum* flowers (Stern, Curry & Whitten, 1986; Borba & Semir, 1998; Teixeira, Borba & Semir, 2004), there are only few (mostly taxonomic) studies that have observed, and sometimes roughly elucidated, the mode of auto-pollination in *Bulbophyllum*, namely in species from South-East Asia (New Guinea, Indonesia, Borneo, Java), Africa, and Madagascar, as well as the adjacent Mascarene Island of La Réunion (Table 1). Based on the few data available, *Bulbophyllum* spp. accomplish auto-pollination through either the lack of a rostellum (e.g. *Bulbophyllum scrobiculilabre* J.J.Sm.), the development of additional anthers contacting the stigma (*Bulbophyllum triandrum* Schltr.) or a stigmatic rostellum (*Bulbophyllum bicoloratum* Schltr.) (Table 1). Notably, there are also early reports of both pollinator-dependent and auto-pollinating individuals in each of two *Bulbophyllum* spp. from New Guinea (Schlechter, 1914; Kirchner, 1922; Smith, 1928), and similar observations have recently been made in the Madagascan endemic *B. bicoloratum* (Gamisch *et al*., 2013). The general lack of large-scale surveys on auto-pollination in orchid genera such as *Bulbophyllum* makes it desirable at this point to elucidate whether, how and to what extent this phenomenon varies within and among groups of closely-related species.

**Table 1:** List of reported auto-pollinating *Bulbophyllum* spp., including mode of auto-pollination, location, conspecific outcrossers, and references

Species	Mode of auto-pollination	Location	Conspecific outcrossers	Reference
*Bulbophyllum aphanopetalum* Schltr.	Absence of rostellum?	New Guinea	–	Schlechter (1907); Kirchner (1922)
*Bulbophyllum cleistogamum* Ridl.	–	Indonesia	–	Smith (1928)
*Bulbophyllum dasyphyllum* Schltr.	–	New Guinea	Yes	Schlechter (1914); Kirchner (1922); Smith (1928)
*Bulbophyllum dischidiifolium* J.J.Sm.	–	Indonesia	–	Smith (1909); Kirchner (1922)
*Bulbophyllum triandrum* Schltr.	Additional anthers contact stigma	New Guinea	–	Smith (1928)
*Bulbophyllum nieuwenhuisii* J.J.Sm.	–	Borneo	–	Smith (1928)
*Bulbophyllum scrobiculilabre* J.J.Sm.	Absence of rostellum	New Guinea	–	Smith (1928)
*Bulbophyllum verruciferum* Schltr. var. *carinatisepalum* Schltr.	–	New Guinea	Yes	Schlechter (1914); Kirchner (1922)
*Bulbophyllum pulvinatum* Schltr.	–	New Guinea	–	Schlechter (1914)
*Bulbophyllum ballii*† P.J.Cribb	–	Africa	–	Vermeulen (1987)
*Bulbophyllum curvimentatum* J.J.Verm.	–	Africa	–	Vermeulen (1987)
*Bulbophyllum oreonastes* Rchb.f.	–	Africa	–	Vermeulen (1987)
*Bulbophyllum occlusum* Ridl.	–	Réunion	Yes*	Jacquemyn *et al*. (2005)
*Bulbophyllum occultum* Thouars	Absence of rostellum*	Madagascar, Réunion	Yes*	Jacquemyn *et al*. (2005); present study
*Bulbophyllum pusillum* (H.Perrier) G.A.Fischer & P.J.Cribb	Absence of rostellum*	Africa, Madagascar, Réunion	Yes*	Jacquemyn *et al*. (2005); present study
*Bulbophyllum erectum*‡ Thouars	Absence of rostellum	Madagascar	Yes*	Schlechter (1924); present study
*Bulbophyllum bicoloratum* Schltr.	Absence of rostellum* and stigmatic rostellum	Madagascar	Yes*,§	Gamisch *et al*. (2013); present study
*Bulbophyllum quadrifarium* Rolfe	Absence of rostellum*	Madagascar	Yes*	Rolfe (1905); present study
*Bulbophyllum obtusatum* Schltr.	Absence of rostellum*	Madagascar	Yes*	Present study
*Bulbophyllum humblotii* Rolfe	Absence of rostellum*	Madagascar, Réunion	Yes*	Present study
*Bulbophyllum complanatum* H.Perrier	Absence of rostellum*	Madagascar	Yes*	Present study

* Information obtained in the present study; –, no information available.

† *Bulbophyllum balli* is considered to be synonymous to *Bulbophyllum pusillum* (J. J. Vermeulen, pers. comm.).

‡ Schlechter (1924) described the auto-pollinating *Bulbophyllum calamarioides* Schltr., a synonym of *Bulbophyllum erectum*.

§ *Bulbophyllum bicoloratum* is trimorphic (see text).

For the present study, we aimed to investigate the floral micromorphology and capacity for autonomous fruit set within and among 29 species of a phylogenetically well-defined lineage of *Bulbophyllum* (termed ‘clade C’) with a centre of distribution in Madagascar and adjacent islands (sections *Calamaria* Schltr., *Humblotiorchis* Schltr., and *Bifalcula* Schltr. *sensu* Fischer, 2007; Fischer *et al*., 2007; Cribb & Hermans, 2009; G. A. Fischer, B. Gravendeel, J. Hermans, A. Sieder, M. Kiehn, J. Andriantiana & P. J. Cribb, unpubl. data). In addition, we extended our surveys of floral morphology to c. 185 accessions (≥ 49 species) of 12 closely allied sections, which, with clade C, form a monophyletic group of taxa predominantly distributed in Madagascar (Fischer, 2007; Fischer *et al*., 2007). To our knowledge, this is the first extensive survey of auto-pollination in a tropical orchid genus. We make a first attempt to discuss the evolutionary implications of our findings, even though the present data only set the stage for further phylogenetic, experimental, and ecological enquiries.

## Material and Methods

### Study system

The focal species of the present study are members of sections *Calamaria*, *Humblotiorchis*, and *Bifalcula* (32 species in total), which form a well-supported subgroup (hereafter ‘clade C’) as part of a largely monophyletic Madagascan *Bulbophyllum* based on recent molecular phylogenetic analyses (Fischer *et al*., 2007; A. Gamisch, G. A. Fischer & H. P. Comes, unpubl. data). Most species of clade C are restricted to Madagascar (27 species) and/or adjacent islands (Mascarenes: La Réunion/Mauritius; Comores; Seychelles; three species), with the remainder being found in Madagascar and/or the East African mainland (*Bulbophyllum humblotii* Rolfe ex Scott-Elliot; *Bulbophyllum malawiense* B.Morris). Their preferential habitats include seasonally dry to humid evergreen forests or, more rarely, marshland, at various elevations (0–1800 m) (Sieder *et al*., 2007; Cribb & Hermans, 2009; G. A. Fischer, B. Gravendeel, J. Hermans, A. Sieder, M. Kiehn, J. Andriantiana & P. J. Cribb, unpubl. data). In the study area, pollinator records for *Bulbophyllum* are limited to La Réunion, where small flies of Drosophilidae and Platystomatidae have been observed visiting the flowers of, respectively, the clade C species *Bulbophyllum incurvum* Thouars (T. Pailler, pers. comm. 2010) and a close relative, the sapromyiophilous *Bulbophyllum variegatum* Thouars (Humeau *et al*., 2011).

**Figure 2:**
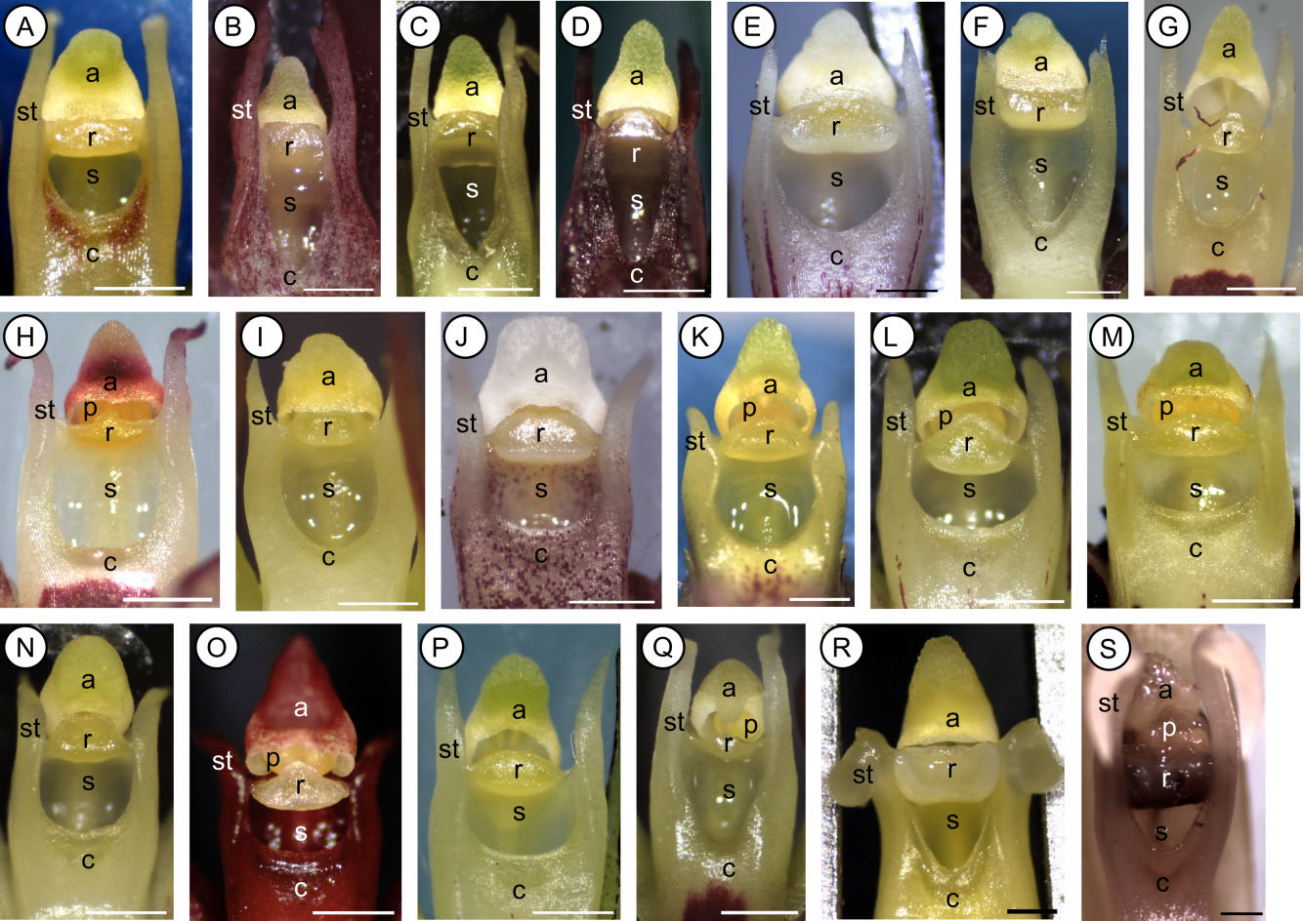
Dissecting images of isolated gynostemia of 19 Madagascan *Bulbophyllum* clade C species showing only the outcrossing (Type I) morph with a distinct (erect) rostellum (Table 2). A, *Bulbophyllum capuronii*. B, *Bulbophyllum* sp. *A*. C, *Bulbophyllum implexum*. D, *Bulbophyllum minutum*. E, *Bulbophyllum histrionicum*. F, *Bulbophyllum ruginosum*. G, *Bulbophyllum elliotii*. H, *Bulbophyllum luteobracteatum*. I, *Bulbophyllum pervillei*. J, *Bulbophyllum trifarium*. K, *Bulbophyllum hildebrandtii*. L, *Bulbophyllum* sp. *E2*. M, *Bulbophyllum malawiense*. N, *Bulbophyllum senghasii*. O, *Bulbophyllum* sp. *C*. P, *Bulbophyllum* sp. *E1*. Q, *Bulbophyllum incurvum*. R, *Bulbophyllum cirrhoglossum*. S, *Bulbophyllum lecouflei*. No images are available of *Bulbophyllum cryptostachium* and *Bulbophyllum rubrum*, which fall into the same category (Table 2). a, anther; c, column; p, pollinia; r, rostellum; s, stigmatic cavity; st, stelidium. Scale bar = 0.5 mm. All photographs by A. Gamisch.

**Figure 3:**
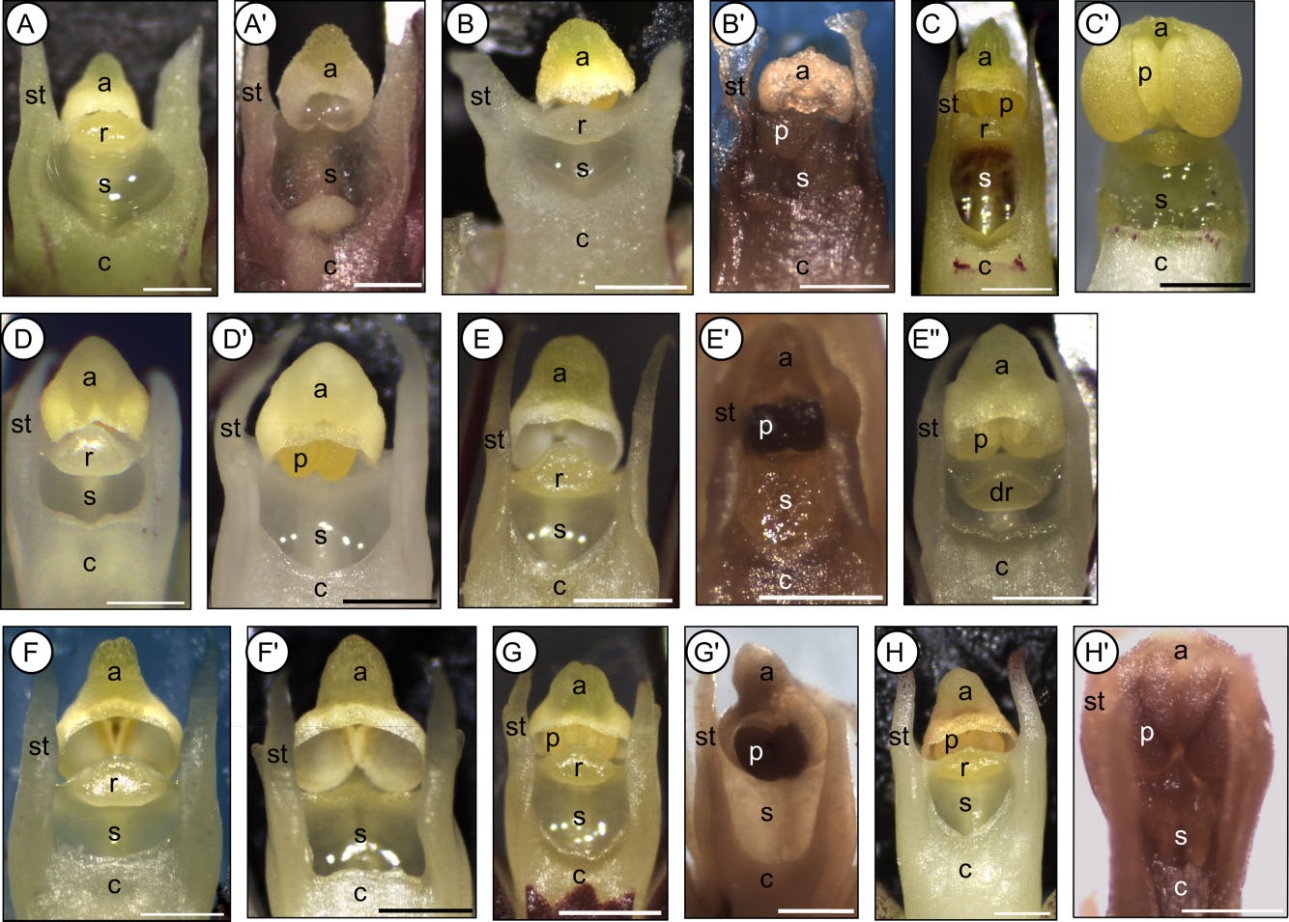
Dissecting images of isolated gynostemia of eight *Bulbophyllum* clade C species showing both outcrossing (Type I) and selfing (Type II/III) morphs. Images are arranged according to species and mating morph. A, A′, *Bulbophyllum complanatum* (Type I and II, respectively). B, B′, *Bulbophyllum humblotii* (I, II). C, C′, *Bulbophyllum erectum* (I, II). D, D′, *Bulbophyllum occultum* (I, II). E, E′, E", *Bulbophyllum bicoloratum* (I, II, III). F, F′, *Bulbophyllum quadrifarium* (I, II). G, G′, *Bulbophyllum pusillum* (I, II). H, H′, *Bulbophyllum obtusatum* (I, II). a, anther; c, column; dr, displaced (sub-erect) rostellum; p, pollinia; r, rostellum; s, stigmatic cavity; st, stelidium. Scale bar = 0.5 mm. All photographs by A. Gamisch. Images E and E″ are modified from Gamisch *et al*. (2013).

**Figure 4:**
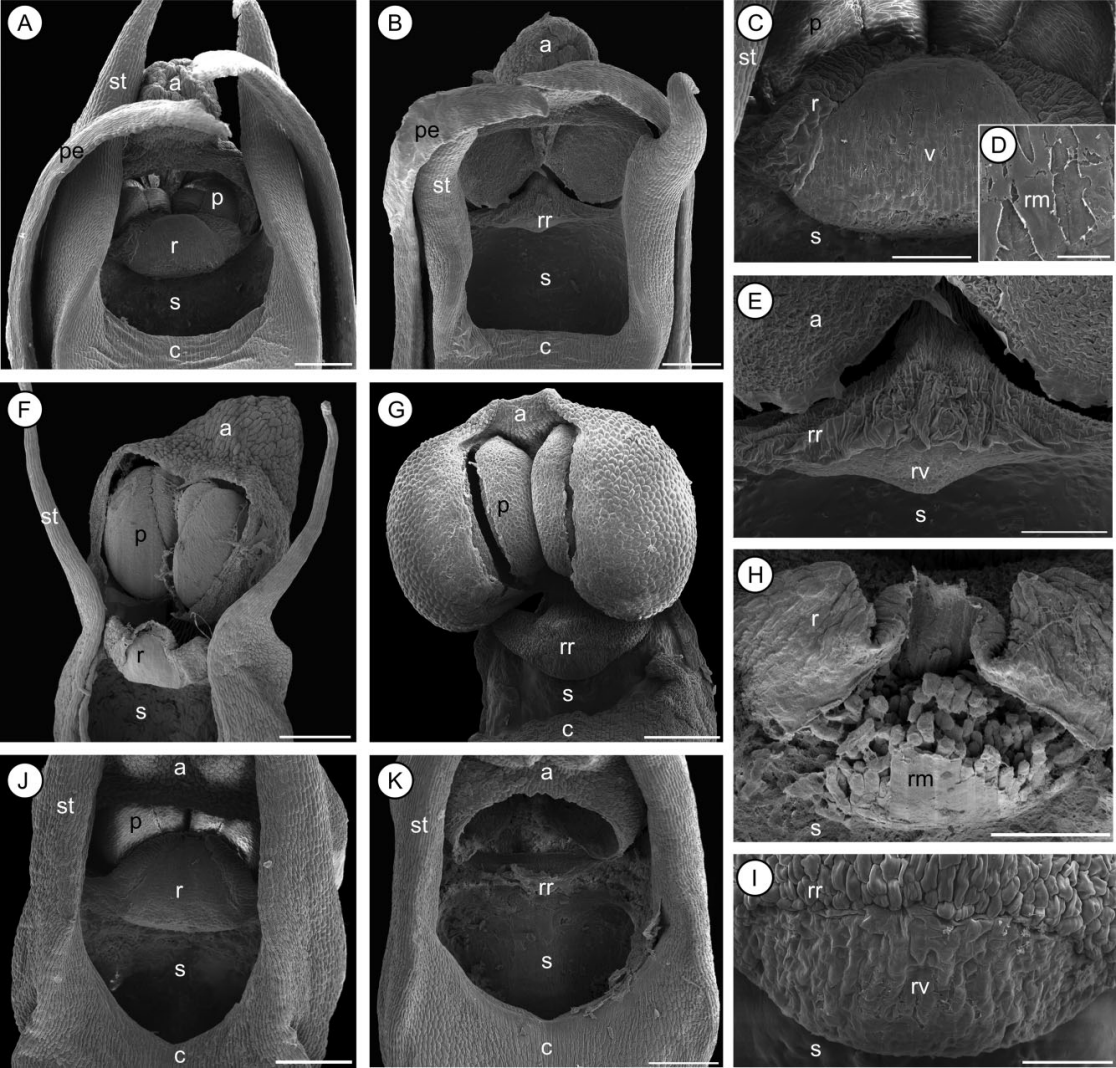
Scanning electron microscopy micrographs of different gynostemium morphs (Types I versus II) observed in *Bulbophyllum quadrifarium* (A, B, C, D, E), *Bulbophyllum erectum* (F, G, H, I), and *Bulbophyllum occultum* (J, K). A, B, gynostemia of *B. quadrifarium* (Type I and II, respectively). C, rostellum of Type I; the central-lateral part has marked epidermal-cuticular foldings, whereas the anterior part is comprised of the half-oval viscidium. D, close-up of the viscidium surface; a smooth, extracellular rostellar membrane covers the viscidium cells. E, rudimentary rostellum and viscidium of Type II. F, G: gynostemia of *B. erectum* (I, II); note the lack of stelidia in G. H, rostellum of Type I; the disrupted rostellar membrane only partly covers the viscidium cells. I, rudimentary rostellum and viscidium of Type II. J, K: gynostemia of *B. occultum* (I, II). a, anther; c, column; p, pollinia; pe, petal; r, rostellum; rm, rostellar membrane; rr, rudimentary rostellum; rv, rudimentary viscidium; s, stigmatic cavity; st, stelidium; v, viscidium. Scale bars = 0.2 mm (A, B, F, G, H); 0.1 mm (C, E, H); 0.01 mm (D); 0.05 mm (I). All micrographs by A. Gamisch and U. Gartner (only F).

There are several floral features that bear on the present study. In general, species of clade C (and their closest relatives) display one or few, many-flowered inflorescences with resupinate, small (between 4 × 3 mm and 10.0 × 7.5 mm) flowers that are characterized by a tongue-shaped, thick, and fleshy labellum (modified median petal) (Fig. 1). This ‘lip’ is elastically hinged at the base of an up-curved, short, and massive gynostemium (column), formed by the union of androecium and gynoecium (Fig. 1). The gynostemium usually bears slender arms or projections (termed ‘stelidia’) on each side, and terminates into a single, two-chambered anther with four, hard, nonfriable pollinia, in two pairs, unequal in size and without appendages. The pollinia inside the anther are usually separated from the deeply concave stigma below by an erect, distinctly protruding rostellum (Figs 2, 3A, B, C, D, E, F, G, H). Its anterior part is comprised of a fleshy, sticky and pad-like structure (Fig. 4C), or ‘viscidium’ (*sensu* Rasmussen, 1985, 1986), which usually serves to attach the pollinia to the body of the pollinator, and is therefore often considered typical for cross-pollination (Darwin, 1862, 1877; van der Pijl & Dodson, 1966; Dressler, 1981; Arditti, 1992; van der Cingel, 2001).

**Table 2:** Results of the micromorphological survey and bagging experiments to assess the gynostemium types (I–III) and capacity for autonomous fruit set (per type) in 29 *Bulbophyllum* clade C species (sections *Calamaria*, *Bifalcula*, *Humblotiorchis*)

			Morphology	Bagging experiments	Gynostemium type			Mean % fruit set (per type)		
Section		Species	Number of individuals/flowers	Number of individuals/outcrossers	I	II	III	I	II	III
*Bifalcula*		*Bulbophyllum capuronii* Bosser	2/4	1/1	2	0	0	0 ± 0	–	–
	*	*Bulbophyllum complanatum* H.Perrier	7/9	6/3	4	3	0	0 ± 0	42.2 ± 5.69	–
		*Bulbophyllum implexum* Jum. & H.Perrier	10/13	4/4	10	0	0	0 ± 0	–	–
		*Bulbophyllum minutum* Thouars	4/4	3/3	4	0	0	0 ± 0	–	–
		*Bulbophyllum* sp. nov. *A*	2/3	2/2	2	0	0	0 ± 0	–	–
*Calamaria*	*	*Bulbophyllum bicoloratum* Schltr.	16/41	13/4	5	1	10	0 ± 0	NA	86.4 ± 10.53†
		*Bulbophyllum cirrhoglossum* H.Perrier	4/7	1/1	4	0	0	0 ± 0	–	–
		*Bulbophyllum cryptostachium* Schltr.	1/1	NA	1	0	0	NA	–	–
		*Bulbophyllum elliotii* Rolfe	4/4	1/1	4	0	0	0 ± 0	–	–
	*	*Bulbophyllum erectum* Thouars	18/49	7/2	5	13	0	0 ± 0	57.7 ± 11.66	–
		*Bulbophyllum hildebrandtii* Rchb.f.	12/21	2/2	12	0	0	0 ± 0	–	–
		*Bulbophyllum histrionicum* G.A.Fischer & P.J.Cribb	7/12	4/4	4	0	0	0 ± 0	–	–
		*Bulbophyllum incurvum* Thouars	4/9	1/1	4	0	0	0 ± 0	–	–
		*Bulbophyllum lecouflei* Bosser	3/3	NA	3	0	0	NA	–	–
		*Bulbophyllum luteobracteatum* Jum. & H.Perrier	4/5	2/2	4	0	0	0 ± 0	–	–
		*Bulbophyllum malawiense* B.Morris	1/5	1/1	1	0	0	1.92 ± 0	–	–
	*	*Bulbophyllum obtusatum* (Jum. & H.Perrier) Schltr.	5/7	1/1	4	1	0	0 ± 0	NA	–
	*	*Bulbophyllum occultum* Thouars	25/38	21/5	5	20	0	0 ± 0	28.0 ± 13.5	–
		*Bulbophyllum pervillei* Rolfe	10/18	4/4	10	0	0	0 ± 0	–	–
	*	*Bulbophyllum pusillum* (H.Perrier) G.A.Fischer & P.J.Cribb	22/38	5/5	10	12	0	0 ± 0	NA	–
	*	*Bulbophyllum quadrifarium* Rolfe	10/30	6/1	3	7	0	0 ± 0	21.02 ± 6.41	–
		*Bulbophyllum rubrum* Jum. & H.Perrier	4/5	NA	4	0	0	NA	–	–
		*Bulbophyllum ruginosum* H.Perrier	3/5	1/1	3	0	0	0 ± 0	–	–
		*Bulbophyllum senghasii* G.A.Fischer & A.Sieder	5/8	5/5	5	0	0	0 ± 0	–	–
		*Bulbophyllum* sp. *C*	2/4	2/2	2	0	0	0 ± 0	–	–
		*Bulbophyllum* sp. *E1*	7/14	2/2	7	0	0	0 ± 0	–	–
		*Bulbophyllum* sp. *E2*	1/4	1/1	1	0	0	0 ± 0	–	–
		*Bulbophyllum trifarium* Rolfe	6/10	3/3	6	0	0	0 ± 0	–	–
*Humblotiorchis*	*	*Bulbophyllum humblotii* Rolfe ex Scott-Elliot	9/10	1/1	6	3	0	8.3 ± 0	NA	–

Indicated for each species: the number of individuals and flowers subjected to the morphological analysis; the number of individuals bagged, both in total and those possessing the ‘outcrossing’ gynostemium type (I); and the number of individuals observed per gynostemium type and their corresponding percentages of fruit set (mean ± SD). NA, not analyzed.

* Species identified to be dimorphic (or trimorphic) for outcrossing (I) and selfing (II/III) types (see text).

† Excluded from this calculation was the fruit set (approximately 25%) of one Type III individual of *Bulbophyllum bicoloratum* (Botanical Garden of Salzburg University collection number FS5709) infested with parasites.

### Plant material

Experimental and micromorphological analyses reported in the present study are based on the following sources, unless stated otherwise: (1) living plants grown at the Botanical Garden University of Salzburg under optimized day/night conditions of temperature (298.15–303.15 K/289.15–291.15 K) and humidity (60–70%/90%); (2) herbarium specimens provided by BR, G, K, MO, P and UPS; and (3) spirit-preserved samples deposited at the Botanical Garden University of Salzburg or loaned from the Botanical Garden University of Vienna, REU and the above mentioned herbaria. Most cultivated and preserved samples are the result of various scientific expeditions (conducted in collaboration between the Botanical Garden University of Salzburg, the Botanical Garden University of Vienna and Parc Botanique et Zoologique de Tsimbazaza) to Madagascar and La Réunion in 2003–2010, with all necessary permits obtained by the Département des Eaux et Fôrets (Madagascar) and the Parc National de La Réunion, thus complying with all relevant regulations.

### Micromorphological and experimental analysis

Micromorphological features of the gynostemium (column) were initially examined in 29 clade C species, represented by either living material (*N* = 106 accessions), spirit-preserved samples (*N* = 78), and/or herbarium vouchers (*N* = 35). Open and pre-anthetic flowers of this material were dissected, observed, and photographed under a Leica EZ4D stereomicroscope (Leica Microsystems). Flowers of herbarium specimens were softened in boiling water prior to dissection. On average, eight individuals per species (mean ± SD: 7.17 ± 6.11) and two flowers per individual (mean ± SD: 1.84 ± 1.63) were examined, resulting in a total of 208 individuals and 382 flowers (Table 2).

To examine further the structural modifications underlying auto-pollination versus pollinator-dependence (see Results), three species (*Bulbophyllum erectum* Thouars, *Bulbophyllum occultum* Thouars, *Bulbophyllum quadrifarium* Rolfe) were chosen for scanning electron microscopy (SEM) studies. For each species, two to five fresh flowers of at least two cultivated individuals differing in gynostemium type were collected, preserved in standard formaldehyde-acetic acid-alcohol (absolute ethanol, 90%; glacial acetic acid, 5%; formaldehyde, 5%), and dissected under a stereomicroscope. The isolated gynostemia were washed, dehydrated through a graded ethanol series, and dried in a Bal-Tec CPD 030 critical point dryer (Bal-Tec AG). The samples were then mounted on aluminium stubs with colloidal carbon, coated with gold using a sputter coater (Agar Scientific) for 90 s, and observed under a Philips XL-30 ESEM scanning electron microscope (FEI Electron Optics) operated at 10–15 kV.

Bagging experiments were performed in the greenhouse at the Botanical Garden University of Salzburg using material of 26 of the 29 clade C species analyzed for gynostemium type (except *Bulbophyllum lecouflei* Bosser, *Bulbophyllum rubrum* Jum. & H.Perrier, *Bulbophyllum cryptostachium* Schltr.) to test for their ability to reproduce by automatic (i.e. vectorless) self-pollination and fruit set. Just prior to anthesis, one inflorescence per plant was enclosed in a bag made of transparent fine-mesh cloth to exclude possible pollinators. Bags were maintained until the end of the fruiting period. On average, four individuals per species (mean ± SD: 3.84 ± 4.35) were bagged (100 individuals in total) (Table 2). Fruit set was quantified for each treatment approximately 1 week after flowers had withered and calculated as the proportion of ripe capsules relative to the total number of flowers per inflorescence (= individual) and averaged across individuals of the same morphotype per species. For one species (*B. occultum*), the ability to reproduce by auto-pollination was also tested under natural conditions in La Réunion (coordinates are available upon request) during the 2010 flowering season. One inflorescence on each of 12 individuals, randomly chosen in the population, was bagged as described above, with fruit set recorded at the end of the growing season. Together, these micromorphological and experimental analyses allowed us to determine the mating type of 29 out of 32 clade C species (208 individuals), except three other still undescribed species (*B.* sp. ‘B’, sp. ‘D’ and sp. ‘F’) (Table 2 see also Supporting information, Table S1).

Because auto-pollination in clade C species is unequivocally reflected in the morphology of the gynostemium (see Results), we aimed to investigate whether such structural features also occur in close relatives. Accordingly, two flowers (mean ± SD: 1.238 ± 0.474) of 185 spirit-preserved *Bulbophyllum* specimens were processed for stereomicroscopy as described above. This extended survey included: (i) 49 extra-clade C species (101 specimens), representing 12 (out of the 16) additional sections of *Bulbophyllum* from the Madagascan region [i.e. sections *Alcistachys* Schltr., *Cirrhopetalum* (Lindl.) Rchb.f., *Elasmotopus* Schltr., *Inversiflorum* G.A.Fischer & P.J.Cribb, *Kainochilus* Schltr., *Lemuraea* Schltr., *Lepiophylax* Schltr., *Lichenophylax* Schltr., *Loxosepalum* Schltr., *Pachychlamys* Schltr., *Pantoblepharon* Schltr., *Ploiarium* Schltr.; Fischer *et al*., 2007; G. A. Fischer, B. Gravendeel, J. Hermans, A. Sieder, M. Kiehn, J. Andriantiana & P. J. Cribb, unpubl. data), and (ii) 84 *Bulbophyllum* accessions from the same region but of unknown species identity (see Supporting information, Table S2). In total, the sampling of the present study comprises 393 *Bulbophyllum* accessions, representing at least 78 species of mainly Madagascan origin (clade C: 29; extra-clade C: 49), and thus approximately 37% of the total species diversity of the genus in this region (approximately 210 species; Fischer *et al*., 2007).

## Results

### Gynostemium micromorphology of clade C species and close relatives

**Figure 5:**
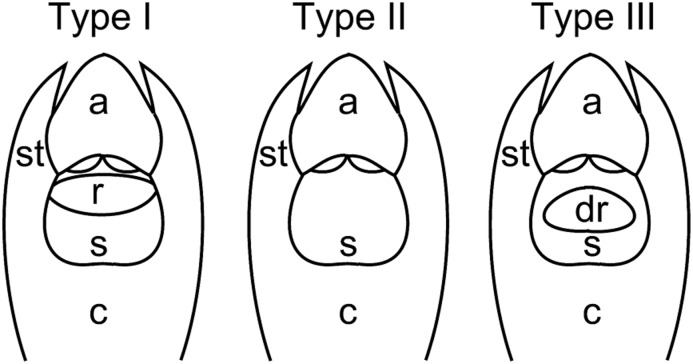
Explanatory sketches of the three gynostemium types identified in Madagascan *Bulbophyllum*. In Type I individuals, an erect and distinctly protruding rostellum separates the pollinia inside the anther from the stigmatic cavity below, ensuring outcrossing. In Type II individuals, the rostellum is practically lacking and apparently unable to act as a physical barrier to prevent selfing. Type III individuals (only *Bulbophyllum bicoloratum*) display a well-developed but sub-erect (‘displaced’) rostellum with stigmatic function facilitating auto-pollination (Gamisch *et al*., 2013). a, anther; c, column; dr, displaced (sub-erect) rostellum; r, rostellum; s, stigmatic cavity; st, stelidium. Drawings by A. Gamisch.

Observations under the stereomicroscope revealed three different types of gynostemium structure (hereafter ‘Types I–III’) among the 29 *Bulbophyllum* clade C species (208 individuals) surveyed (Figs 2, 3, 4, 5, Table 2). Type I samples (*N* = 138) displayed the conventional, erect, and distinctly protruding rostellum that separates the pollinia inside the anther from the stigmatic cavity (Figs 2, 3A, B, C, D, E, F, G, H). In Type II individuals (*N* = 60), this rostellum was practically absent and just visible as a small, thin margin, apparently unable to act as a physical barrier (Fig. 3A′, B′, C′, D′, E′, F′, G′, H′). However, SEM observations in three species (*B. erectum*, *B. occultum*, and *B. quadrifrarium*) demonstrated that this margin still bears features typical of a well-developed rostellum (i.e. a central-lateral part with marked epidermal-cuticular foldings and an anterior part comprised of a smooth, half-oval to triangular viscidium-like structure) (Fig. 4E, I, ‘rr’ and ‘rv’, respectively). Finally, Type III individuals (*N* = 10) displayed a well-developed but sub-erect (‘displaced’) rostellum, which was only found in a single species, *B. bicoloratum*, as described previously (Fig. 3E") (Gamisch *et al*., 2013).

The occurrence of Types I–III, however, differed within and among the 29 clade C species (Table 2). For 21 of those, we obtained evidence of only Type I individuals. In seven species, however, two morphs (I and II) were observed, and, in *B. bicoloratum*, three morphs (I–III). Most of these di- or trimorphic species are members of the species-rich section *Calamaria* [*B. bicoloratum*, *B. erectum*, *Bulbophyllum obtusatum* Schltr., *B. occultum*, *Bulbophyllum pusillum* (H.Perrier) G.A. Fischer & P.J.Cribb, *B. quadrifarium*], except *Bulbophyllum complanatum* H.Perrier (section *Bifalcula*) and *B. humblotii* (section *Humblotiorchis*). Notably, in neither instance of gynostemium type variation was there any evidence of intra-individual variation (i.e. all flowers within a given individual displayed the same type, regardless of whether pre-anthetic or newly opened flowers were examined) (A. Gamisch, pers. observ.). Furthermore, we did not observe any gross morphological differences in vegetative or floral phenotype between individuals differing in gynostemium type within a given species. Thus, apart from their subtle differences in the structure, position, and/or function of the rostellum, the gynostemia of Type I–III conspecifics were more-or-less identical in almost all dimorphic (or trimorphic) species. These similarities also concern: (1) the presence of stelidia; (2) the shape of the stigmatic cavity (triangular, ovoid or crescent; depending on the species); and (3) the production of copious stigmatic exudate in both pre-anthetic and newly opened flowers (Figs 2, 3, 4). A remarkable exception, however, involved *B. erectum*, in which Type II individuals were found to differ from their Type I conspecifics in the lack of stelidia (compare Fig. 4F, G), fewer flowers per inflorescence and flowers that opened less wide (A. Gamisch, pers. observ.). All the spirit-preserved material of extra-clade C *Bulbophyllum* surveyed (185 specimens in total) revealed only individuals possessing the conventional (Type I) rostellum (see Supporting information, Table S1).

### Mating system of clade C species: bagging experiments, and fruit set

Twenty-six of the 29 clade C species analyzed for gynostemium type (100 individuals) were subjected to bagging experiments under greenhouse conditions to test for their ability of auto-pollination (Table 2; see also Supporting information, Table S1). All individuals possessing a well-developed, erect rostellum (Type I) failed to set any fruit, except one Type I individual each of *B. humblotii* and *B. malawiense*, which nonetheless had low fruit set (8% and 2%, respectively). By contrast, fruit set in individuals essentially lacking a rostellum (Type II) tended to be high, with per-species means ranging from 21% to 57%, and an overall mean of 37%. In *B. bicoloratum*, the exceptionally high fruit set of Type III individuals (86%) is readily explained by the stigmatic function of their sub-erect rostellum facilitating auto-pollination as reported previously (Gamisch *et al*., 2013). Although seed production was not measured, self-fertilized capsules generally contained an abundance of seeds. When tested for *B. occultum* (Type II) and *B. bicoloratum* (Type III), these seeds proved viable based on tetrazolium staining (U. Jaros, unpubl. data).

## Discussion

The transition from outcrossing to self-fertilization is considered the most common evolutionary transition in flowering plants, and it has occurred repeatedly in many independent lineages, including those of Orchidaceae (Catling, 1990; Wright, Kalisz & Slotte, 2013). Moreover, the absence of a rostellum is by far the most common mechanism of auto-pollination in this family, and is found in approximately half the self-pollinating orchid species studied so far (Catling, 1990; Tałałaj & Brzosko, 2008; Peter & Johnson, 2009; Zhou *et al*., 2012; Suetsugu, 2013). Several observations from the present study, as summarized and discussed below, tend to be consistent with these previous generalizations and allow for some tentative evolutionary conclusions on the direction, development, relative timing, and adaptive significance of mating type shifts in *Bulbophyllum*.

### Recurrent mating type polymorphism in *Bulbophyllum* clade C species

The observational and experimental results of the present study suggest that individuals of *Bulbophyllum* clade C species may be readily grouped as to mating system based on three kinds of structural modifications involving the gynostemium, namely the rostellum (Types I–III; Table 2; for explanatory sketches, see Fig. 5). Thus, the inability of auto-pollination, as assessed by fruit set failure in bagged flowers, was almost unequivocally expressed by the presence of a well-developed, erect rostellum (Type I), which is often considered typical for insect-mediated cross-pollination in orchids (Dressler, 1981; Arditti, 1992; van der Cingel, 2001; Tan & Nishida, 2005). By contrast, the ability of auto-pollination was most often related to the practical absence of a rostellum (Type II), thus permitting unhindered contact between pollinia and stigmatic fluid. In some rare instances, however, auto-pollination was facilitated through the presence of a sub-erect, stigmatic rostellum (Type III; only *B. bicoloratum*), allowing the penetration of pollen tubes from pollinia *in situ* (Gamisch *et al*., 2013).

However, there is one notable exception to the above patterns: two out of 62 Type I individuals examined showed some (but low) autonomous fruit set (≤ 8%), suggesting that pollinia may occasionally slide down onto the stigmatic surface, bypassing the rostellum. This, however, is probably a casual and quantitatively insignificant process compared to the far more efficient auto-pollination mechanism of Type II and III individuals, in which autonomous fruit set on average was much higher (37% and 86%, respectively), in keeping with the range of fruit set observed in other auto-pollinating orchids (14–100%; Tremblay *et al*., 2005). Furthermore, it is important to emphasize that the three gynostemium types identified varied among (but not within) individuals, in which they were consistently expressed in (pre-)anthetic flowers. Hence, there was no evidence for rostellum disintegration during ontogeny as sometimes observed in other auto-pollinating orchids (Catling, 1990; Peter & Johnson, 2009). Even though the precise genetic control and heritability of Types I–III remain obscure, it must be presumed that they are entirely genetically encoded, and possibly unresponsive to environmental influences. We therefore consider individuals of Types II and III as genetically fixed auto-pollinators (‘selfers’), whereas those of Type I represent the conventional pollinator-dependent form (‘outcrossers’).

The most significant finding to emerge from the present study is the apparent co-existence of two (or rarely three) discrete gynostemium types within eight out of the 29 species of clade C surveyed (Fig. 3, Table 2). Specifically, seven species of this group were found to be dimorphic for outcrossing (Type I) and selfing (Type II) morphs, whereas, in *B. bicoloratum*, all three types (I–III) were observed. Overall, this exemplifies another striking incidence of polymorphic mating type variation within orchid species, which often involves developmental and structural modifications of the gynostemium (Catling, 1990). However, we are only aware of dimorphic cases, such as *Cypripedium passerinum* Richardson, in which the pollinia and stigma of selfing plants develop in close contact (Catling, 1990; Catling & Bennett, 2007), whereas, in *Spiranthes ovalis* Lindl., the selfing variant (var. *erostellata* Catling) lacks a rostellum (Catling, 1983), as reported in the present study for Type II morphs of Madagascan *Bulbophyllum*.

With often small sample sizes within species, caution must be applied in interpreting the present data because future sampling may reveal auto-pollinating variants in those remaining clade C species classified here as outcrossers (Type I). Despite this limitation, it is intriguing that all 185 extra-clade C specimens of *Bulbophyllum* surveyed (≥ 49 species, mainly from Madagascar) displayed only the conventional outcrossing (I) morph (see Supporting information, Table S2). If we assume that the frequency of selfers and outcrossers within extra-clade C follows a binomial distribution, it is possible to calculate the number of selfers expected for this group based on the outcrosser (I) to selfer (II/III) ratio observed within clade C (148 : 70) (Bennett & Husby, 2008). Accordingly, we would expect to see between 51 and 74 selfing morphs (*P* = 0.99) in our extra-clade C sample, instead of none (A. Gamisch, unpubl. data). We therefore tentatively conclude that the frequency of selfers in this latter sample is unlikely to be as high as within clade C, even though further sampling is required to test this hypothesis. Nonetheless, all of the currently available data suggest that auto-pollination is a relatively rare phenomenon in *Bulbophyllum* from the Madagascan region, where it appears to be largely associated (for yet unknown reasons) with certain species of this particular clade, and exclusively as part of an intraspecific polymorphism in mating type (Table 1). The evolutionary implications of these findings are discussed below.

### Direction of mating type evolution

The conventional morph (Type I) occurs in all examined species of clade C, and is probably the common, if not prevalent one, among its close relatives. Thus, it is likely that outcrossing represents the ancestral character state of clade C. However, it still remains unclear whether auto-pollination as a result of rostellum loss (Type II), as observed in eight polymorphic species, has evolved only once in their common ancestor (and subsequently been retained as shared ancestral (I/II) polymorphism) or independently on many occasions. We tend to favour this latter hypothesis because this kind of auto-pollination is found in each of the three sections of clade C and because this presence/absence character may well have a simple genetic basis, favouring rapid and parallel trait evolution (Gottlieb, 1984; Coyne & Lande, 1985; Rieseberg & Burke, 2001; Lankinen, 2009). To distinguish between these competing (single versus multiple origin) hypotheses, work is underway to generate a sufficiently resolved molecular phylogenetic tree of clade C for use in ancestral character state reconstructions. However, even at this point, there can be little doubt that auto-pollination via rostellum receptivity (Type III) represents a uniquely derived character state of *B. bicoloratum*, in which it most probably evolved from an erect (nonreceptive) rostellum, implying a shift in the function of a pre-existing trait or ‘exaptation’ (Gamisch *et al*., 2013).

### Developmental mode of rostellum loss

Of particular note is the existence of a rudimentary rostellum-viscidium in selfing (Type II) individuals, as most clearly revealed by our SEM observations on *B. quadrifarium* (Fig. 4B, E) and *B. erectum* (Fig. 4G, I). This is of relevance considering renewed interest in the temporal alterations of developmental pathways leading to evolutionary change in morphology (‘heterochrony’; Gould, 1977; Alberch *et al*., 1979; Box & Glover, 2010; Rudall, Perl & Bateman, 2013). Indeed, there have been few similar detailed (e.g. micromorphological, SEM) studies in Orchidaceae comparing gynostemium differences between auto-pollinators and their close relatives, and those that did have not found such a vestigial organ (e.g. in *Epipactis flaminia* P.R.Savelli & Aless.: Bonatti, Sgarbi & Del Prete, 2006). Hence, to our knowledge, this is the first documented case of a rudimentary rostellum-viscidium in selfing orchids, whereby this structure could have been easily overlooked at the macroscopic level in this and earlier studies (Fig. 3) (Catling, 1990; Micheneau *et al*., 2008). Its existence clearly provides further support for the derived character state of selfing (Type II) in clade C species. Moreover, it testifies to the commonly held notion that self-fertilization in orchids (as in many other flowering plants) has often evolved through heterochronic–paedomorphic modification of outcrossing flowers (Richards, 1982; Ehlers & Pedersen, 2000; Bonatti *et al*., 2006), implying the retention of a juvenile feature (here of the rostellum) of the ancestor into mature individuals of the descendant. Even though such developmental shifts remain poorly understood (Rudall *et al*., 2013), paedomorphosis is often viewed an important basis for rapid evolutionary transitions toward simpler morphologies associated with self-fertilization (Ehlers & Pedersen, 2000; Box & Glover, 2010; Li & Johnston, 2010; but see also Armbruster *et al*., 2013). An implication of this is the possibility that transitions toward auto-pollination in clade C species occurred recurrently by paedomorphosis through a retarded (neotenic), abbreviated (progenetic) and/or delayed (post-displaced) development of the ‘rostellum’ in Type II selfers compared with their outcrossing conspecifics (for terminology, see also Box & Glover, 2010).

### Relatively recent shifts in mating system?

In orchids, as in other flowering plants, it has long been recognized that transitions from outbreeding to selfing are often accompanied by a characteristic set of changes to the morphology and function of vegetative organs and, in particular, of flowers, together termed the ‘selfing syndrome’ (e.g. reduced flower size, dull flower coloration, lack of nectar or scent, cleistogamy, phyllanthy; Darwin, 1876; Ornduff, 1969; Richards, 1982; Catling, 1990; Sicard & Lenhard, 2011). Especially in short-lived plants, such changes may occur rapidly (e.g. on millennial times scales; Foxe *et al*., 2009) and be driven by selection for more efficient self-pollination (Sicard & Lenhard, 2011). However, our present observations provide only limited evidence for such a selfing syndrome in *Bulbophyllum* clade C. A notable exception is *B. erectum*, in which auto-pollinating (Type II) individuals were found to differ from outcrossing conspecifics both in several attraction traits (i.e. reduced flower size and number; A. Gamisch, pers. observ.) and the lack of stelidia (Figs 3C′, 4G). These column arms may well qualify as a mechanical-fit trait (sensu Nattero, Cocucci & Medel, 2010) because they usually force the pollinator to adopt a position that results in very precise pollinia transfer (Jones & Gray, 1976). However, because none of the above features is associated with the process of auto-pollination, their modifications (after the transition to selfing) most probably result from a reallocation of resources (Sicard & Lenhard, 2011) and/or a lack of pollinator-mediated selection for attractiveness and mechanical fit (Catling, 1990; Nattero *et al*., 2010). Otherwise, the essential lack of a selfing syndrome in *Bulbophyllum* clade C supports our principal hypothesis that shifts toward auto-pollination in this group occurred relatively recently. Nonetheless, it would be interesting to compare selfers and outcrossers within and among species of this group in terms of potentially more ‘subtle’ trait differences in fragrance, nectar production, lip-osmophore density or ovule number and development (Tremblay *et al*., 2005; Micheneau *et al*., 2008; Wiemer *et al*., 2009).

### Potential adaptive significance of auto-pollination

A final question arises about the selective factor(s) that could have led to the origin and maintenance of mating type polymorphism, namely selfing/outcrossing in *Bulbophyllum* clade C. In this context, it is worth recalling the two main but mutually non-exclusive models about the short-term selective advantages of selfing in flowering plants (Charlesworth, 2006; Cheptou, 2012; Wright *et al*., 2013). First, as proposed since Darwin (1862, 1877), selfing can be an adaptive, reproductive assurance strategy in response to low mate and/or pollinator availability (e.g. as a consequence of habitat fragmentation) and may result in increased colonizing ability (Hagerup, 1952; Jain, 1976; Lloyd, 1979; Williamson, 1984; Catling, 1990; Eckert *et al*., 2010; but see also Busch *et al*., 2011). Second, as known since Fisher (1941), a plant genotype that can both self-fertilize and disperse pollen benefits from a 50% transmission advantage over either an obligate outcrosser or an obligate selfer (Cheptou, 2012; Pettengill & Moeller, 2012; Wright *et al*., 2013). However, there are at least two observations emerging from the present study that tend to reject this latter hypothesis for the evolution of selfing in *Bulbophyllum* clade C. First, in type II selfers, the practical absence of a viscidium renders both the export and receipt of pollinia an inaccurate and inefficient, if not improbable affair (Hollingsworth *et al*., 2006; Armbruster & Muchhala, 2009). Second, in cultivated material of each selfing variant (II, III), we observed pollinia to slide down into the stigmatic cavity almost immediately after anthesis; if this holds true in nature, pollinia would be unavailable for siring offspring on other individuals. Taken together, we consider that both selfing variants are unlikely to reproduce through outcrossed pollinia, and thus most often behave as obligate rather than partial selfers. Consequently, any such selfing mutant arising in an obligate outcrossing population would be unable to spread ‘automatically’ by a 50% transmission advantage of its (selfing) genes over those of the outcrossers (Jarne & Charlesworth, 1993; Holsinger, 2000). We therefore infer that, in Madagascan *Bulbophyllum* clade C, adaptive selection has favoured the origin and maintenance of auto-pollination that ensures reproduction when pollinators and/or mates are rare or absent. The catalysts initiating such environmental conditions remain elusive at this point. However, it is tempting to speculate that human-mediated deforestation and degradation, which has so severely impacted the primary forest in Madagascar, especially since the 1950/70s (Burney *et al*., 2004; Harper *et al*., 2007; Cable, 2011), had a decisive role. Such effects of human-modified landscapes on plant mating patterns have been studied thoroughly (Eckert *et al*., 2010) and typically manifest as increased selfing for self-compatible species (Breed *et al*., 2012). It is feasible, therefore, that the eight *Bulbophyllum* clade C species that are polymorphic for mating type may well be on an evolutionary trajectory toward increased selfing.

### Conclusions

Based on 393 *Bulbophyllum* accessions, representing approximately 37% of the species diversity of the genus in the Madagascan region, this micromorphological and experimental study identified eight species of a single lineage (‘clade C’) in which auto-pollination usually involves the practical absence of a rostellum or, rarely, the presence of a sub-erect, stigmatic rostellum. These findings are mainly in agreement with earlier studies reporting relatively frequent loss of the rostellum in selfing, albeit mostly terrestrial, extra-tropical orchids. However, a novel and unexpected finding was that in each of those eight species, auto-pollinating morphs (selfers) coexist with their pollinator-dependent conspecifics (outcrossers), possessing the conventional erect (nonreceptive) rostellum. Although further research is required (e.g. phylogenetic, ecological, genetic–developmental), we hypothesize that auto-pollination via rostellum abortion has a simple genetic basis, and probably evolved rapidly, and perhaps multiple times, as a result of subtle changes in the timing of rostellum development (heterochrony). Thus, species of clade C may have an intrinsic genetical and developmental lability toward auto-pollination, allowing fast evolutionary response under environmental, perhaps human-disturbed conditions favouring reproductive assurance. Future studies need to investigate how these gynostemium morphs evolved over time, space, and the environment, as well as how their frequencies vary within and among populations experiencing different degrees of habitat fragmentation. This should not only provide additional insights on how these mating type polymorphisms are maintained in present-day populations of *Bulbophyllum* clade C species, but also improve our understanding of how tropical orchids adapt to changing environmental conditions.

## Supporting Information

**Table S1.** List of 208 individual accessions of 29 *Bulbophyllum* clade C species (sects. *Calamaria*, *Bifalcula*, *Humblotiorchis*) from the Madagascan region, including information on the Botanical Garden University of Salzburg (HBS) or Vienna (HBV) cultivation and/or voucher numbers, sources, number of flowers surveyed for gynostemium structure, treatment in bagging experiments, identified gynostemium (‘morph’) type, and inferred mating type. AP, auto-pollinating; CP, cross-pollinating; fresh, fresh material; herb, herbarium-derived; spirit, spirit-preserved. **Table S2.** List of 185 individual, spirit-preserved accessions of *Bulbophyllum* extra-clade C material from the Madagascan region (12 sections), including information on voucher numbers, sources, distribution and collection area (indicated by an asterisk), and the number of flowers surveyed for gynostemium structure, resulting only in Type I (see text). Note that this list includes 84 specimens of unknown species identity.
