# Supplementary material for: Recurrent polymorphic mating type variation in Madagascan Bulbophyllum species (Orchidaceae) exemplifies a high incidence of auto-pollination in tropical orchids
Source: Bot J Linn Soc. 2014 May 20;175(2):242–58. doi: 10.1111/boj.12168 (PMC4373168; doi:10.1111/boj.12168)
Supplement: Supplementary file 1 — Table S1. List of 208 individual accessions of 29 Bulbophyllum clade C species (sects. Calamaria, Bifalcula, Humblotiorchis) from the Madagascan region, including information on the Botanical Garden University of Salzburg (HBS) or Vienna (HBV) cultivation and/or voucher numbers, sources, number of flowers surveyed for gynostemium structure, treatment in bagging experiments, identified gynostemium (‘morph’) type, and inferred mating type. AP, auto-pollinating; CP, cross-pollinating; fresh, fresh material; herb, herbarium-derived; spirit, spirit-preserved. Table S2. List of 185 individual, spirit-preserved accessions of Bulbophyllum extra-clade C material from the Madagascan region (12 sections), including information on voucher numbers, sources, distribution and collection area (indicated by an asterisk), and the number of flowers surveyed for gynostemium structure, resulting only in Type I (see text). Note that this list includes 84 specimens of unknown species identity. [file boj0175-0242-sd1.doc]

SUPPORTING INFORMATION

**Table S1**. List of 208 individual accessions of 29 *Bulbophyllum* clade C species (sects. *Calamaria*, *Bifalcula,* *Humblotiorchis*) from the Madagascan region, including information on HBS cultivation and/or voucher numbers, sources, number of flowers surveyed for gynostemium structure, treatment in bagging experiments, identified gynostemium (‘morph’) type, and inferred mating type. Abbreviations: AP, auto-pollinating; CP, cross-pollinating; fresh, fresh material; herb, herbarium-derived; spirit, spirit-preserved.

| Section | Species | Voucher  number | | Institution of deposition | | Collectors | | No. of flowers per individual | | | | | | Bagging experiment | | | Morph type | | Mating type | |  | |
| --- | --- | --- | --- | --- | --- | --- | --- | --- | --- | --- | --- | --- | --- | --- | --- | --- | --- | --- | --- | --- | --- | --- |
|  |  |  | |  | |  | | fresh | spirit | herb | | total | |  | | |  | |  | |  | |
| *Calamaria* | *B. bicoloratum* Schltr. | FS5852 | | HBS | | G. Fischer, A. Sieder & J. Andriantiana | | 1 |  |  | | 1 | | x | | | I | | CP | |  | |
|  |  | FS1052 | | HBS | | G. Fischer, A. Sieder & J. Andriantiana | | 1 | 5 |  | | 6 | | x | | | I | | CP | |  | |
|  |  | FS5697 | | HBS | | G. Fischer, A. Sieder & J. Andriantiana | | 1 |  |  | | 1 | | x | | | III | | AP | |  | |
|  |  | FS5832 | | HBS | | G. Fischer, A. Sieder & J. Andriantiana | | 2 |  |  | | 2 | | x | | | III | | AP | |  | |
|  |  | FS5849 | | HBS | | G. Fischer, A. Sieder & J. Andriantiana | | 2 |  |  | | 2 | | x | | | I | | CP | |  | |
|  |  | FS5686 | | HBS | | G. Fischer, A. Sieder & J. Andriantiana | | 3 |  |  | | 3 | | x | | | III | | AP | |  | |
|  |  | FS5695 | | HBS | | G. Fischer, A. Sieder & J. Andriantiana | | 3 |  |  | | 3 | | x | | | III | | AP | |  | |
|  |  | FS5687 | | HBS | | G. Fischer, A. Sieder & J. Andriantiana | | 2 | 4 |  | | 6 | | x | | | III | | AP | |  | |
|  |  | FS5696 | | HBS | | G. Fischer, A. Sieder & J. Andriantiana | | 3 |  |  | | 3 | | x | | | III | | AP | |  | |
|  |  | FS5710 | | HBS | | G. Fischer, A. Sieder & J. Andriantiana | | 3 |  |  | | 3 | | x | | | III | | AP | |  | |
|  |  | FS5709 | | HBS | | G. Fischer, A. Sieder & J. Andriantiana | | 3 |  |  | | 3 | | x | | | III | | AP | |  | |
|  |  | FS5684 | | HBS | | G. Fischer, A. Sieder & J. Andriantiana | | 2 |  |  | | 2 | | x | | | III | | AP | |  | |
|  |  | FS807 | | HBS | | G. Fischer, A. Sieder & J. Andriantiana | |  | 1 |  | | 1 | | x | | | I | | CP | |  | |
|  |  | FS2118 | | HBS | | G. Fischer & J. Andriantiana | |  | 2 |  | | 2 | |  | | | I | | CP | |  | |
|  |  | FS5469 | | HBS | | G. Fischer & J. Andriantiana | |  | 2 |  | | 2 | |  | | | III | | AP | |  | |
|  |  | FS1035 | | HBS | | G. Fischer, A. Sieder & J. Andriantiana | |  | 1 |  | | 1 | |  | | | II | | AP | |  | |
| *Bifalcula* | *B. capuronii* Bosser | FS1010 | | HBS | | G. Fischer, A. Sieder & J. Andriantiana | | 2 | 1 |  | | 3 | | x | | | I | | CP | |  | |
|  |  | 5990519 | | MO | | - | |  |  | 1 | | 1 | |  | | | I | | CP | |  | |
| *Calamaria* | *B. cirrhoglossum* H.Perrier | FS4125 | | HBS | | A. Sieder & J. Andriantiana | | 3 | 1 |  | | 4 | | x | | | I | | CP | |  | |
|  |  | 3801670 | | MO | | - | |  |  | 1 | | 1 | |  | | | I | | CP | |  | |
|  |  | P00097484 | | P | | Kiener | |  |  | 1 | | 1 | |  | | | I | | CP | |  | |
|  |  | P00097483 | | P | | H. Perrier de la Bâthie | |  | 1 |  | | 1 | |  | | | I | | CP | |  | |
| *Bifalcula* | *B. complanatum* H.Perrier | FS5762 | | HBS | | G. Fischer, A. Sieder & J. Andriantiana | | 1 |  |  | | 1 | | x | | | I | | CP | |  | |
|  |  | FS5762 | | HBS | | G. Fischer, A. Sieder & J. Andriantiana | | 1 |  |  | | 1 | | x | | | I | | CP | |  | |
|  |  | FS5779 | | HBS | | G. Fischer, A. Sieder & J. Andriantiana | | 2 |  |  | | 2 | | x | | | II | | AP | |  | |
|  |  | FS5866 | | HBS | | G. Fischer, A. Sieder & J. Andriantiana | | 2 |  |  | | 2 | | x | | | II | | AP | |  | |
|  |  | P00097213 | | P | | H. Humbert & R. Capuron | |  | 1 |  | | 1 | |  | | | I | | CP | |  | |
|  |  | FS5762 | | HBS | | G. Fischer, A. Sieder & J. Andriantiana | | 1 |  |  | | 1 | | x | | | I | | CP | |  | |
|  |  | FS5797 | | HBS | | G. Fischer, A. Sieder & J. Andriantiana | | 1 |  |  | | 1 | | x | | | II | | AP | |  | |
| *Calamaria* | *B. cryptostachium* Schltr. | P00097524 | | P | | H. Perrier de la Bâthie | |  |  | 1 | | 1 | |  | | | I | | CP | |  | |
| *Calamaria* | *B. elliotii* Rolfe | FS863 | | HBS | | G. Fischer, A. Sieder & J. Andriantiana | |  | 1 |  | | 1 | |  | | | I | | CP | |  | |
|  |  | FS5448 | | HBS | | G. Fischer & J. Andriantiana | | 1 |  |  | | 1 | |  | | | I | | CP | |  | |
|  |  | OR1164 | | HBS | | - | | 1 |  |  | | 1 | |  | | | I | | CP | |  | |
|  |  | FS5918 | | HBS | | G. Fischer, A. Sieder & J. Andriantiana | | 1 |  |  | | 1 | | x | | | I | | CP | |  | |
| *Calamaria* | *B. erectum* Thouars | FS861 | | HBS | | G. Fischer, A. Sieder & J. Andriantiana | | 2 |  |  | | 2 | | x | | | II | | AP | |  | |
|  |  | FS5778 | | HBS | | G. Fischer, A. Sieder & J. Andriantiana | | 1 |  |  | | 1 | | x | | | I | | CP | |  | |
|  |  | FS4047 | | HBS | | A. Sieder & J. Andriantiana | | 5 |  |  | | 5 | | x | | | II | | AP | |  | |
|  |  | FS1023 | | HBS | | G. Fischer, A. Sieder & J. Andriantiana | | 1 |  |  | | 1 | | x | | | II | | AP | |  | |
|  |  | FS1020 | | HBS | | G. Fischer, A. Sieder & J. Andriantiana | | 8 | 7 |  | | # | |  | | | II | | AP | |  | |
|  |  | FS5906 | | HBS | | G. Fischer, A. Sieder & J. Andriantiana | | 2 |  |  | | 2 | | x | | | II | | AP | |  | |
|  |  | FS5383 | | HBS | | G. Fischer & J. Andriantiana | | 7 |  |  | | 7 | | x | | | I | | CP | |  | |
|  |  | FS4203 | | HBS | | G. Fischer & J. Andriantiana | |  | 2 |  | | 2 | |  | | | II | | AP | |  | |
|  |  | FS5268 | | HBS | | G. Fischer & J. Andriantiana | |  | 1 |  | | 1 | |  | | | II | | AP | |  | |
|  |  | FS4230 | | HBS | | G. Fischer & J. Andriantiana | |  | 1 |  | | 1 | |  | | | II | | AP | |  | |
|  |  | FS1054 | | HBS | | G. Fischer, A. Sieder & J. Andriantiana | |  | 1 |  | | 1 | |  | | | II | | AP | |  | |
|  |  | FS5966 | | HBS | | G. Fischer, A. Sieder & J. Andriantiana | |  | 1 |  | | 1 | |  | | | II | | AP | |  | |
|  |  | FS6049 | | HBS | | G. Fischer, A. Sieder & J. Andriantiana | |  | 1 |  | | 1 | |  | | | II | | AP | |  | |
|  |  | FS1021 | | HBS | | G. Fischer, A. Sieder & J. Andriantiana | |  | 3 |  | | 3 | |  | | | I | | CP | |  | |
|  |  | FS797 | | HBS | | G. Fischer, A. Sieder & J. Andriantiana | |  | 3 |  | | 3 | |  | | | I | | CP | |  | |
|  |  | 5990514 | | MO | | - | |  |  | 1 | | 1 | |  | | | II | | AP | |  | |
|  |  | P0102799 | | P | | H. Perrier de la Bâthie | |  |  | 1 | | 1 | |  | | | I | | CP | |  | |
|  |  | FS4140 | | HBS | | A. Sieder & J. Andriantiana | | 1 |  |  | | 1 | | x | | | II | | AP | |  | |
| *Calamaria* | *B. hildebrandtii* Rchb.f. | FS5648 | | HBS | | G. Fischer, A. Sieder & J. Andriantiana | | 1 |  |  | | 1 | | x | | | I | | CP | |  | |
|  |  | FS5744 | | HBS | | G. Fischer, A. Sieder & J. Andriantiana | | 1 |  |  | | 1 | |  | | | I | | CP | |  | |
|  |  | OR1360 | | HBS | | - | | 3 |  |  | | 3 | | x | | | I | | CP | |  | |
|  |  | FS1169 | | HBS | | G. Fischer, A. Sieder,  W. Knirsch & J. Andriantiana | |  | 3 |  | | 3 | |  | | | I | | CP | |  | |
|  |  | FS1157 | | HBS | | G. Fischer, A. Sieder,  W. Knirsch & J. Andriantiana | |  | 2 |  | | 2 | |  | | | I | | CP | |  | |
|  |  | FS1133 | | HBS | | G. Fischer, A. Sieder,  W. Knirsch & J. Andriantiana | |  | 2 |  | | 2 | |  | | | I | | CP | |  | |
|  |  | FS1151 | | HBS | | G. Fischer, A. Sieder,  W. Knirsch & J. Andriantiana | |  | 1 |  | | 1 | |  | | | I | | CP | |  | |
|  |  | FS1286 | | HBS | | G. Fischer, A. Sieder,  W. Knirsch & J. Andriantiana | |  | 2 |  | | 2 | |  | | | I | | CP | |  | |
|  |  | FS5725 | | HBS | | G. Fischer, A. Sieder & J. Andriantiana | |  | 2 |  | | 2 | |  | | | I | | CP | |  | |
|  |  | FS6067 | | HBS | | G. Fischer, A. Sieder & J. Andriantiana | |  | 2 |  | | 2 | |  | | | I | | CP | |  | |
|  |  | UPS V-45802, 33036 | | UPS | | - | |  |  | 1 | | 1 | |  | | | I | | CP | |  | |
|  |  | FS1225 | | HBS | | G. Fischer, A. Sieder,  W. Knirsch & J. Andriantiana | |  |  | 1 | | 1 | |  | | | I | | CP | |  | |
| *Calamaria* | *B. histrionicum* G. A.Fischer & P.J.Cribb | FS5899 | | HBS | | G. Fischer, A. Sieder & J. Andriantiana | | 1 |  |  | | 1 | | x | | | I | | CP | |  | |
|  |  | FS6059 | | HBS | | G. Fischer, A. Sieder & J. Andriantiana | | 2 |  |  | | 2 | | x | | | I | | CP | |  | |
|  |  | FS6060 | | HBS | | G. Fischer, A. Sieder & J. Andriantiana | | 2 |  |  | | 2 | | x | | | I | | CP | |  | |
|  |  | OR15 | | HBS | | - | | 1 |  |  | | 1 | | x | | | I | | CP | |  | |
|  |  | FS1232 | | HBS | | G. Fischer, A. Sieder,  W. Knirsch & J. Andriantiana | |  | 3 |  | | 3 | |  | | | I | | CP | |  | |
|  |  | PR733 | | G | | P. Ranirison | |  |  | 1 | | 1 | |  | | | I | | CP | |  | |
|  |  | K1126 | | K | | - | |  | 2 |  | | 2 | |  | | | I | | CP | |  | |
| *Humblotiorchis* | *B. humblotii* Rolfe ex Scott-Elliot | FS841 | | HBS | | G. Fischer, A. Sieder & J. Andriantiana | | 1 |  |  | | 1 | | x | | | I | | CP | |  | |
|  |  | OR262 | | HBS | | - | | 1 |  |  | | 1 | |  | | | I | | CP | |  | |
|  |  | FS1008 | | HBS | | G. Fischer, A. Sieder & J. Andriantiana | |  | 2 |  | | 2 | |  | | | I | | CP | |  | |
|  |  | FS2716 | | HBS | | G. Fischer & J. Andriantiana | |  | 1 |  | | 1 | |  | | | II | | AP | |  | |
|  |  | FS4417 | | HBS | | G. Fischer & J. Andriantiana | |  | 1 |  | | 1 | |  | | | II | | AP | |  | |
|  |  | 5069683 | | MO | | - | |  |  | 1 | | 1 | |  | | | I | | CP | |  | |
|  |  | FS2732 | | HBS | | G. Fischer & J. Andriantiana | |  |  | 1 | | 1 | |  | | | II | | AP | |  | |
|  |  | UPS V45775, 33009 | | UPS | | B. Pettersson & L.A. Nilsson | |  |  | 1 | | 1 | |  | | | I | | CP | |  | |
|  |  | FS1279 | | HBS | | G. Fischer, A. Sieder,  W. Knirsch & J. Andriantiana | |  |  | 1 | | 1 | |  | | | I | | CP | |  | |
| *Bifalcula* | *B. implexum* Jum. & H.Perrier | FS6042 | | HBS | | G. Fischer, A. Sieder & J. Andriantiana | | 1 |  |  | | 1 | | x | | | I | | CP | |  | |
|  |  | FS6042 | | HBS | | G. Fischer, A. Sieder & J. Andriantiana | | 2 |  |  | | 2 | | x | | | I | | CP | |  | |
|  |  | OR1600 | | HBS | | - | | 1 |  |  | | 1 | | x | | | I | | CP | |  | |
|  |  | FS6042 | | HBS | | G. Fischer, A. Sieder & J. Andriantiana | | 1 |  |  | | 1 | | x | | | I | | CP | |  | |
|  |  | FS6042 | | HBS | | G. Fischer, A. Sieder & J. Andriantiana | |  | 2 |  | | 2 | |  | | | I | | CP | |  | |
|  |  | FS1207 | | HBS | | G. Fischer, A. Sieder,  W. Knirsch & J. Andriantiana | |  | 1 |  | | 1 | |  | | | I | | CP | |  | |
|  |  | FS5433 | | HBS | | G. Fischer & J. Andriantiana | |  | 2 |  | | 2 | |  | | | I | | CP | |  | |
|  |  | FS5432 | | HBS | | G. Fischer & J. Andriantiana | |  | 1 |  | | 1 | |  | | | I | | CP | |  | |
|  |  | FS3223 | | HBS | | A. Sieder, W. Knirsch & J. Andriantiana | |  | 1 |  | | 1 | |  | | | I | | CP | |  | |
|  |  | FS5758 | | HBS | | G. Fischer, A. Sieder & J. Andriantiana | |  | 1 |  | | 1 | |  | | | I | | CP | |  | |
| *Calamaria* | *B. incurvum* Thouars | FS1081 | | HBS | | - | | 2 | 3 |  | | 5 | | x | | | I | | CP | |  | |
|  |  | REU10096 | | REU | | T. Pailler | |  | 2 |  | | 2 | |  | | | I | | CP | |  | |
|  |  | REU10095 | | REU | | T. Pailler | |  | 1 |  | | 1 | |  | | | I | | CP | |  | |
|  |  | REU10097 | | REU | | T. Pailler | |  | 1 |  | | 1 | |  | | | I | | CP | |  | |
| *Calamaria* | *B. lecouflei* Bosser | FS1278 | | HBS | | G. Fischer, A. Sieder,  W. Knirsch & J. Andriantiana | |  | 1 |  | | 1 | |  | | | I | | CP | |  | |
|  |  | H2006/00529 | | K | | - | |  |  | 1 | | 1 | |  | | | I | | CP | |  | |
|  |  | P00097482 | | P | | M. Lecoufle | |  |  | 1 | | 1 | |  | | | I | | CP | |  | |
| *Calamaria* | *B. luteobracteatum* Jum. & H.Perrier | FS5512 | | HBS | | G. Fischer & J. Andriantiana | | 1 |  |  | | 1 | | x | | | I | | CP | |  | |
|  |  | FS5512 | | HBS | | G. Fischer & J. Andriantiana | | 2 |  |  | | 2 | | x | | | I | | CP | |  | |
|  |  | FS5501 | | HBS | | G. Fischer & J. Andriantiana | |  | 1 |  | | 1 | |  | | | I | | CP | |  | |
|  |  | P00097749 | | P | | H. Perrier de la Bâthie | |  |  | 1 | | 1 | |  | | | I | | CP | |  | |
| *Calamaria* | *B. malawiense* B.Morris | OR146 | | HBS | | - | | 3 | 2 |  | | 5 | | x | | | I | | CP | |  | |
| *Bifalcula* | *B. minutum* Thouars | FS5916 | | HBS | | G. Fischer, A. Sieder & J. Andriantiana | | 1 |  |  | | 1 | | x | | | I | | CP | |  | |
|  |  | FS5306 | | HBS | | G. Fischer & J. Andriantiana | | 1 |  |  | | 1 | | x | | | I | | CP | |  | |
|  |  | FS5306 | | HBS | | G. Fischer & J. Andriantiana | | 1 |  |  | | 1 | | x | | | I | | CP | |  | |
|  |  | FS1006 | | HBS | | G. Fischer, A. Sieder & J. Andriantiana | |  | 1 |  | | 1 | |  | | | I | | CP | |  | |
| *Calamaria* | *B. obtusatum* (Jum. & H.Perrier) Schltr. | FS5736 | | HBS | | G. Fischer, A. Sieder & J. Andriantiana | | 2 |  |  | | 2 | | x | | | I | | CP | |  | |
|  |  | FS1170 | | HBS | | G. Fischer, A. Sieder,  W. Knirsch & J. Andriantiana | |  | 2 |  | | 2 | |  | | | I | | CP | |  | |
|  |  | P00097472 | | P | | R. Decary | |  |  | 1 | | 1 | |  | | | I | | CP | |  | |
|  |  | P00097473 | | P | | H. Perrier de la Bâthie | |  |  | 1 | | 1 | |  | | | II | | AP | |  | |
|  |  | H2006/00529, 2128 | | K | | - | |  |  | 1 | | 1 | |  | | | I | | CP | |  | |
| *Calamaria* | *B. occultum* Thouars | FS611 | | HBS | | G. Fischer, A. Sieder & J. Andriantiana | | 2 | 1 |  | | 3 | | x | | | II | | AP | |  | |
|  |  | FS617 | | HBS | | G. Fischer, A. Sieder & J. Andriantiana | | 3 | 4 |  | | 7 | | x | | | II | | AP | |  | |
|  |  | FS3813 | | HBS | | W. Knirsch & J. Adriantiana | | 1 | 1 |  | | 2 | | x | | | II | | AP | |  | |
|  |  | FS5935 | | HBS | | G. Fischer, A. Sieder & J. Andriantiana | | 2 |  |  | | 2 | | x | | | I | | CP | |  | |
|  |  | FS5934 | | HBS | | G. Fischer, A. Sieder & J. Andriantiana | | 1 |  |  | | 1 | | x | | | I | | CP | |  | |
|  |  | FS5945 | | HBS | | G. Fischer, A. Sieder & J. Andriantiana | | 1 |  |  | | 1 | |  | | | II | | AP | |  | |
|  |  | FS5290 | | HBS | | G. Fischer & J. Andriantiana | | 1 |  |  | | 1 | | x | | | II | | AP | |  | |
|  |  | FS5037 | | HBS | | G. Fischer & J. Andriantiana | | 1 |  |  | | 1 | | x | | | I | | CP | |  | |
|  |  | FS5028 | | HBS | | G. Fischer & J. Andriantiana | | 1 |  |  | | 1 | | x | | | I | | CP | |  | |
|  |  | FS5054 | | HBS | | G. Fischer & J. Andriantiana | | 1 |  |  | | 1 | | x | | | I | | CP | |  | |
|  |  | FS5040 | | HBS | | G. Fischer & J. Andriantiana | |  | 4 |  | | 4 | |  | | | II | | AP | |  | |
|  |  | BR-S.P. 921 134 | | BR | | - | |  |  | 1 | | 1 | |  | | | II | | AP | |  | |
|  |  | 2747595 | | MO | | - | |  |  | 1 | | 1 | |  | | | II | | AP | |  | |
|  |  | RU_ML1_01_2010 | | field study (Réunion) | | U. Jaros & A. Gamisch | | 1 |  |  | | 1 | | x | | | II | | AP | |  | |
|  |  | RU_ML1_04_2010 | | field study (Réunion) | | U. Jaros & A. Gamisch | | 1 |  |  | | 1 | | x | | | II | | AP | |  | |
|  |  | RU_ML1_12_2010 | | field study (Réunion) | | U. Jaros & A. Gamisch | | 1 |  |  | | 1 | | x | | | II | | AP | |  | |
|  |  | RU_ML1_14_2010 | | field study (Réunion) | | U. Jaros & A. Gamisch | | 1 |  |  | | 1 | | x | | | II | | AP | |  | |
|  |  | RU_ML1_17_2010 | | field study (Réunion) | | U. Jaros & A. Gamisch | | 1 |  |  | | 1 | | x | | | II | | AP | |  | |
|  |  | RU_ML1_18_2010 | | field study (Réunion) | | U. Jaros & A. Gamisch | | 1 |  |  | | 1 | | x | | | II | | AP | |  | |
|  |  | RU_ML2_04_2010 | | field study (Réunion) | | U. Jaros & A. Gamisch | | 1 |  |  | | 1 | | x | | | II | | AP | |  | |
|  |  | RU_ML2_06_2010 | | field study (Réunion) | | U. Jaros & A. Gamisch | | 1 |  |  | | 1 | | x | | | II | | AP | |  | |
|  |  | RU_ML2_07_2010 | | field study (Réunion) | | U. Jaros & A. Gamisch | | 1 |  |  | | 1 | | x | | | II | | AP | |  | |
|  |  | RU_ML2_08_2010 | | field study (Réunion) | | U. Jaros & A. Gamisch | | 1 |  |  | | 1 | | x | | | II | | AP | |  | |
|  |  | RU_ML2_09_2010 | | field study (Réunion) | | U. Jaros & A. Gamisch | | 1 |  |  | | 1 | | x | | | II | | AP | |  | |
|  |  | RU_BB1_06_2010 | | field study (Réunion) | | U. Jaros & A. Gamisch | | 1 |  |  | | 1 | | x | | | II | | AP | |  | |
| *Calamaria* | *B. pervillei* Rolfe | FS818 | | HBS | | G. Fischer, A. Sieder & J. Andriantiana | | 5 | 1 |  | | 6 | | x | | | I | | CP | |  | |
|  |  | S295/96 | | HBV | | - | |  | 2 |  | | 2 | |  | | | I | | CP | |  | |
|  |  | FS1060 | | HBS | | G. Fischer, A. Sieder,  W. Knirsch & J. Andriantiana | |  | 1 |  | | 1 | |  | | | I | | CP | |  | |
|  |  | REU04806 | | REU | | J. Fournel | |  | 3 |  | | 3 | |  | | | I | | CP | |  | |
|  |  | 5553156 | | MO | | - | |  |  | 1 | | 1 | |  | | | I | | CP | |  | |
|  |  | K000410083 | | K | | - | |  |  | 1 | | 1 | |  | | | I | | CP | |  | |
|  |  | P00097515 | | P | | H. Perrier de la Bâthie | |  |  | 1 | | 1 | |  | | | I | | CP | |  | |
|  |  | FS933 | | HBS | | G. Fischer, A. Sieder & J. Andriantiana | | 1 |  |  | | 1 | | x | | | I | | CP | |  | |
|  |  | FS696 | | HBS | | G. Fischer, A. Sieder & J. Andriantiana | | 1 |  |  | | 1 | | x | | | I | | CP | |  | |
|  |  | FS5406 | | HBS | | G. Fischer & J. Andriantiana | | 1 |  |  | | 1 | | x | | | I | | CP | |  | |
| *Calamaria* | *B. pusillum* (H.Perrier) G.A Fischer & P.J.Cribb | FS5719 | | HBS | | G. Fischer, A. Sieder & J. Andriantiana | | 1 |  |  | | 1 | | x | | | I | | CP | |  | |
|  |  | FS5718 | | HBS | | G. Fischer, A. Sieder & J. Andriantiana | | 2 |  |  | | 2 | | x | | | I | | CP | |  | |
|  |  | FS5743 | | HBS | | G. Fischer, A. Sieder & J. Andriantiana | | 1 |  |  | | 1 | | x | | | I | | CP | |  | |
|  |  | FS5720 | | HBS | | G. Fischer, A. Sieder & J. Andriantiana | | 1 |  |  | | 1 | | x | | | I | | CP | |  | |
|  |  | FS3207 | | HBS | | A. Sieder, W. Knirsch & J. Andriantiana | |  | 1 |  | | 1 | |  | | | II | | AP | |  | |
|  |  | S O00B172-1 | | HBS | | A. Sieder & J. Andriantiana | |  | 1 |  | | 1 | |  | | | I | | CP | |  | |
|  |  | FS5717 | | HBS | | G. Fischer, A. Sieder & J. Andriantiana | |  | 2 |  | | 2 | |  | | | II | | AP | |  | |
|  |  | FS5918 | | HBS | | G. Fischer, A. Sieder & J. Andriantiana | |  | 1 |  | | 1 | |  | | | II | | AP | |  | |
|  |  | FS2229 | | HBS | | G. Fischer & J. Andriantiana | |  | 5 |  | | 5 | |  | | | II | | AP | |  | |
|  |  | FS2019 | | HBS | | G. Fischer & J. Andriantiana | |  | 4 |  | | 4 | |  | | | I | | CP | |  | |
|  |  | FS2107 | | HBS | | G. Fischer & J. Andriantiana | |  | 3 |  | | 3 | |  | | | II | | AP | |  | |
|  |  | REU04802 | | REU | | C. Misandeau | |  | 1 |  | | 1 | |  | | | II | | AP | |  | |
|  |  | FS2124 | | HBS | | G. Fischer & J. Andriantiana | |  | 2 |  | | 2 | |  | | | I | | CP | |  | |
|  |  | K61101 | | K | | - | |  | 2 |  | | 2 | |  | | | II | | AP | |  | |
|  |  | FS2281 | | HBS | | G. Fischer & J. Andriantiana | |  | 2 |  | | 2 | |  | | | II | | AP | |  | |
|  |  | REU10100 | | REU | | T. Pailler | |  | 1 |  | | 1 | |  | | | II | | AP | |  | |
|  |  | FS2141 | | HBS | | G. Fischer & J. Andriantiana | |  | 1 |  | | 1 | |  | | | II | | AP | |  | |
|  |  | K55115 | | K | | B. Pettersson & L.A. Nilsson | |  | 1 |  | | 1 | |  | | | II | | AP | |  | |
|  |  | P00097490 | | P | | H. Perrier de la Bâthie | |  |  | 1 | | 1 | |  | | | I | | CP | |  | |
|  |  | P00097491 | | P | | H. Perrier de la Bâthie | |  |  | 1 | | 1 | |  | | | I | | CP | |  | |
|  |  | P00097494 | | P | | H. Perrier de la Bâthie | |  |  | 1 | | 1 | |  | | | II | | AP | |  | |
|  |  | FS5513 | | HBS | | G. Fischer & J. Andriantiana | | 3 |  |  | | 3 | | x | | | I | | CP | |  | |
| *Calamaria* | *B. quadrifarium* Rolfe | FS827 | | HBS | | G. Fischer, A. Sieder & J. Andriantiana | | 6 | 1 |  | | 7 | | x | | | II | | AP | |  | |
|  |  | FS830 | | HBS | | G. Fischer, A. Sieder & J. Andriantiana | | 3 |  |  | | 3 | | x | | | II | | AP | |  | |
|  |  | FS826 | | HBS | | G. Fischer, A. Sieder & J. Andriantiana | | 6 |  |  | | 6 | | x | | | II | | AP | |  | |
|  |  | OR259 | | HBS | | G. Fischer, A. Sieder & J. Andriantiana | | 1 |  |  | | 1 | | x | | | II | | AP | |  | |
|  |  | FS1049 | | HBS | | G. Fischer, A. Sieder & J. Andriantiana | | 9 |  |  | | 9 | | x | | | I | | CP | |  | |
|  |  | FS1051 | | HBS | | G. Fischer, A. Sieder & J. Andriantiana | |  | 1 |  | | 1 | |  | | | II | | AP | |  | |
|  |  | 5990509 | | MO | | - | |  |  | 1 | | 1 | |  | | | I | | CP | |  | |
|  |  | 4988083 | | MO | | - | |  |  | 1 | | 1 | |  | | | II | | AP | |  | |
|  |  | P00097486 | | P | | R. Decary | |  |  | 1 | | 1 | |  | | | I | | CP | |  | |
|  |  | FS825 | | HBS | | G. Fischer, A. Sieder & J. Andriantiana | |  |  |  | | 1 | | x | | | II | | AP | |  | |
| *Calamaria* | *B. rubrum* Jum. & H.Perrier | FS6044 | | HBS | | G. Fischer, A. Sieder & J. Andriantiana | |  | 1 |  | | 1 | |  | | | I | | CP | |  | |
|  |  | 3523007 | | MO | | - | |  |  | 1 | | 1 | |  | | | I | | CP | |  | |
|  |  | P00097762 | | P | | H. Perrier de la Bâthie | |  |  | 2 | | 2 | |  | | | I | | CP | |  | |
|  |  | P00097502 | | P | | H. Perrier de la Bâthie | |  |  | 1 | | 1 | |  | | | I | | CP | |  | |
| *Calamaria* | *B. ruginosum* H.Perrier | FS5885 | | HBS | | G. Fischer, A. Sieder & J. Andriantiana | | 2 |  |  | | 2 | | x | | | I | | CP | |  | |
|  |  | FS5885 | | HBS | | G. Fischer, A. Sieder & J. Andriantiana | |  | 2 |  | | 2 | |  | | | I | | CP | |  | |
|  |  | P00097770 | | P | | H. Perrier de la Bâthie | |  |  | 1 | | 1 | |  | | | I | | CP | |  | |
| *Calamaria* | *B. senghasii* G.A.Fischer & A.Sieder | FS3969 | | HBS | | O. Pronk | | 2 |  |  | | 2 | | x | | | I | | CP | |  | |
|  | |
|  |  | FS3969 | | HBS | | O. Pronk | | 3 |  |  | | 3 | | x | | | I | | CP | |  | |
|  |  | FS3969 | | HBS | | O. Pronk | | 1 |  |  | | 1 | | x | | | I | | CP | |  | |
|  |  | OR1247 | | HBS | | - | | 1 |  |  | | 1 | | x | | | I | | CP | |  | |
|  |  | FS3970 | | HBS | | A. Sieder & J. Andriantiana | | 1 |  |  | | 1 | | x | | | I | | CP | |  | |
| *Bifalcula* | *B. sp. nov. A* | FS2707 | | HBS | | G. Fischer & J. Andriantiana | | 1 |  |  | | 1 | | x | | | I | | CP | |  | |
|  |  | FS5921 | | HBS | | G. Fischer, A. Sieder & J. Andriantiana | | 2 |  |  | | 2 | | x | | | I | | CP | |  | |
| *Calamaria* | *B. sp.nov. C* | FS5383 | | HBS | | G. Fischer & J. Andriantiana | | 3 |  |  | | 3 | | x | | | I | | CP | |  | |
|  |  | FS5246 | | HBS | | G. Fischer & J. Andriantiana | | 1 |  |  | | 1 | | x | | | I | | CP | |  | |
| *Calamaria* | *B. sp. nov. E1* | FS6022 | | HBS | | G. Fischer, A. Sieder & J. Andriantiana | | 2 |  |  | | 2 | | x | | | I | | CP | |  | |
|  |  | FS6023 | | HBS | | G. Fischer, A. Sieder & J. Andriantiana | | 4 |  |  | | 4 | | x | | | I | | CP | |  | |
|  |  | FS6027 | | HBS | | G. Fischer, A. Sieder & J. Andriantiana | | 1 |  |  | | 1 | |  | | | I | | CP | |  | |
|  |  | FS6028 | | HBS | | G. Fischer, A. Sieder & J. Andriantiana | | 1 |  |  | | 1 | |  | | | I | | CP | |  | |
|  |  | FS6022 | | HBS | | G. Fischer, A. Sieder & J. Andriantiana | |  | 2 |  | | 2 | |  | | | I | | CP | |  | |
|  |  | FS6033 | | HBS | | G. Fischer, A. Sieder & J. Andriantiana | |  | 2 |  | | 2 | |  | | | I | | CP | |  | |
|  |  | FS6030 | | HBS | | G. Fischer, A. Sieder & J. Andriantiana | |  | 2 |  | | 2 | |  | | | I | | CP | |  | |
| *Calamaria* | *B. sp. nov. E2* | FS5797 | | HBS | | G. Fischer, A. Sieder & J. Andriantiana | | 4 |  |  | | 4 | | x | | | I | | CP | |  | |
| *Calamaria* | *B. trifarium* Rolfe | FS5628 | | HBS | | G. Fischer, A. Sieder & J. Andriantiana | | 1 |  |  | | 1 | | x | | | I | | CP | |  | |
|  |  | OR1576 | | HBS | | - | | 3 |  |  | | 3 | | x | | | I | | CP | |  | |
|  |  | FS1224 | | HBS | | G. Fischer, A. Sieder,  W. Knirsch & J. Andriantiana | |  | 3 |  | | 3 | |  | | | I | | CP | |  | |
|  |  | FS5630 | | HBS | | G. Fischer, A. Sieder & J. Andriantiana | |  | 1 |  | | 1 | |  | | | I | | CP | |  | |
|  |  | FS5632 | | HBS | | G. Fischer, A. Sieder & J. Andriantiana | | 1 |  |  | | 1 | | x | | | I | | CP | |  | |
|  |  | P00097496 | | P | | R. Decary | |  |  | 1 | | 1 | |  | | | I | | CP | |  | |
|  |  |  |  | |  | |  | | | |  | |  | |  |  | |  | |  | |  |

**Table S2.** List of 185 individual, spirit-preserved accessions of *Bulbophyllum* extra-clade C material from the Madagascan region (12 sections), including information on voucher numbers, sources, distribution and collection area (indicated by an asterisk), and the number of flowers surveyed for gynostemium structure, resulting only in Type I (see text). Note that this list includes 84 specimens of unknown species identity.

| Section | Species | Voucher number | Institution of deposition | Collectors | Distribution and collection area | No. of flowers |
| --- | --- | --- | --- | --- | --- | --- |
|
| *Alcistachys* | *B. bathieanum* Schltr. | FS5162 | HBS | G. Fischer & J. Andriantiana | Madagascar* | 2 |
|  |  | FS5024 | HBS | G. Fischer & J. Andriantiana | Madagascar* | 2 |
|  |  | FS5069 | HBS | G. Fischer & J. Andriantiana | Madagascar* | 1 |
|  |  | FS5025 | HBS | G. Fischer & J. Andriantiana | Madagascar* | 2 |
|  |  | Birkinshaw1301 | MO | C. Birkinshaw | Madagascar* | 1 |
| *Alcistachys* | *B. brevipetalum* H.Perrier | FS2048 | HBS | G. Fischer & J. Andriantiana | Madagascar* | 1 |
|  |  | Buerki105 | MO | S. Buerki, C. Rakotovao & M. Callmander | Madagascar* | 2 |
| *Alcistachys* | *B. hamelini* W.Watson | FS3257 | HBS | A. Sieder, W. Knirsch & J. Andriantiana | Madagascar* | 1 |
|  |  | K73999 | K | - | Madagascar* | 1 |
| *Alcistachys* | *B. occlusum* Ridl. | OR999 | HBS | - | Madagascar*, Réunion | 2 |
|  |  | SO00B270-1 | HBV | A. Sieder & J. Andriantiana | Madagascar*, Réunion | 2 |
|  |  | FS1501 | HBS | G. Fischer, A. Sieder, W. Knirsch & J. Andriantiana | Madagascar*, Réunion | 2 |
|  |  | SO00B375-1 | HBV | A. Sieder & J. Andriantiana | Madagascar*, Réunion | 1 |
|  |  | M786 | K | D.J. Du Puy, P. Cribb & J. Andriantiana | Madagascar*, Réunion | 2 |
|  |  | K10742 | K | - | Madagascar*, Réunion | 1 |
| *Alcistachys* | *B. sp.* | SO00B34-1 | HBV | A. Sieder & J. Andriantiana | Madagascar* | 2 |
| *Alcistachys* | *B. sp.* | FS2972 | HBS | A. Sieder, W. Knirsch & J. Andriantiana | Madagascar* | 3 |
| *Alcistachys* | *B. sp.* | FS4391 | HBS | G. Fischer & J. Andriantiana | Madagascar* | 2 |
| *Alcistachys* | *B. sulfureum* Schltr. | JH4900 | K | J. Hermans | Madagascar* | 1 |
| *Alcistachys* | *B. variegatum* Thouars | K7156/1662 | K | L.M. Mason | Madagascar, Réunion, Mauritius, Comores | 2 |
|  |  | REU10387 | REU | J. Fournel | Madagascar, Réunion*, Mauritius, Comores | 1 |
|  |  | FS5976 | HBS | G. Fischer, A. Sieder & J. Andriantiana | Madagascar*, Réunion, Mauritius, Comores | 2 |
|  |  | FS799 | HBS | G. Fischer, A. Sieder & J. Andriantiana | Madagascar*, Réunion, Mauritius, Comores | 2 |
|  |  | HBSOR23 | HBS | - | Madagascar*, Réunion, Mauritius, Comores | 1 |
| *Cirrhopetalum* | *B. longiflorum* Thouars | FS5000 | HBS | G. Fischer & J. Andriantiana | Madagascar*, Malawie, Mauritius, New Caledonia, Papua New Guinea, Phillipines, Réunion, Samoa; Seychelles; Solomon Island, Uganda, Tanzania, Vanuatu, Vietnam, Zimbabwe | 1 |
| *Elasmotopus* | *B. sp.* | FS4729 | HBS | A. Sieder, W. Knirsch, Ch. Berg. & M. Pinter | Madagascar* | 1 |
| *Inversiflorum* | *B. cardiobulbum* Bosser | FS5139 | HBS | G. Fischer & J. Andriantiana | Madagascar* | 1 |
|  |  | FS5140 | HBS | G. Fischer & J. Andriantiana | Madagascar* | 1 |
| *Inversiflorum* | *B. uroplatoides* Hermans & G.A.Fischer | FS5056 | HBS | G. Fischer & J. Andriantiana | Madagascar* | 1 |
|  |  | FS5133 | HBS | G. Fischer & J. Andriantiana | Madagascar* | 1 |
|  |  | FS5055 | HBS | G. Fischer & J. Andriantiana | Madagascar* | 1 |
| *Kainochilus* | *B. alexandrae* Schltr. | FS1367 | HBS | G. Fischer, A. Sieder, W. Knirsch & J. Andriantiana | Madagascar* | 3 |
| *Kainochilus* | *B. edentatum* H.Perrier | FS866 | HBS | G. Fischer, A. Sieder & J. Andriantiana | Madagascar* | 2 |
| *Kainochilus* | *B. imerinense* Schltr. | FS2783 | HBS | G. Fischer & J. Andriantiana | Madagascar* | 3 |
|  |  | FS2991 | HBS | A. Sieder, W. Knirsch & J. Andriantiana | Madagascar* | 2 |
|  |  | FS2892 | HBS | G. Fischer & J. Andriantiana | Madagascar* | 2 |
| *Kainochilus* | *B. sp.* | FS5554 | HBS | G. Fischer & J. Andriantiana | Madagascar* | 1 |
| *Kainochilus* | *B. viguieri* Schltr | FS2580 | HBS | G. Fischer & J. Andriantiana | Madagascar* | 2 |
| *Lemuraea* | *B. nutans* Thouars | FS969 | HBS | G. Fischer, A. Sieder & J. Andriantiana | Madagascar*, Réunion, Mauritius | 1 |
|  |  | FS2245 | HBS | G. Fischer & J. Andriantiana | Madagascar*, Réunion, Mauritius | 1 |
|  |  | FS2084 | HBS | G. Fischer & J. Andriantiana | Madagascar*, Réunion, Mauritius | 1 |
| *Lepiophylax* | *B. conchidioides* Ridl. | FS1327 | HBS | G. Fischer, A. Sieder, W. Knirsch & J. Andriantiana | Madagascar* | 1 |
|  |  | FS2835 | HBS | G. Fischer & J. Andriantiana | Madagascar* | 1 |
|  |  | FS1921 | HBS | G. Fischer & J. Andriantiana | Madagascar* | 1 |
| *Lepiophylax* | *B. jumelleanum* Schltr. | FS5791 | HBS | G. Fischer, A. Sieder & J. Andriantiana | Madagascar* | 1 |
| *Lepiophylax* | *B. sp.* | FS3197 | HBS | A. Sieder, W. Knirsch & J. Andriantiana | Madagascar* | 1 |
| *Lichenophylax* | *B. debile* Bosser | FS5546 | HBS | G. Fischer & J. Andriantiana | Madagascar* | 1 |
| *Lichenophylax* | *B. forsythianum* Kraenzl. | FS1552 | HBS | G. Fischer, A. Sieder, W. Knirsch & J. Andriantiana | Madagascar* | 1 |
| *Lichenophylax* | *B. sp.* | H2010_00322#2 | K | - | Madagascar* | 1 |
| *Lichenophylax* | *B. sp.* | H2010_00322#4 | K | - | Madagascar* | 1 |
| *Lichenophylax* | *B. sp.* | FS2224 | HBS | G. Fischer & J. Andriantiana | Madagascar* | 1 |
| *Lichenophylax* | *B. sp.* | FS3882 | HBS | W. Knirsch & J. Adriantiana | Madagascar* | 1 |
| *Lichenophylax* | *B. sp.* | FS2204 | HBS | A. Sieder & J. Andriantiana | Madagascar* | 1 |
| *Lichenophylax* | *B. sp.* | FS5529 | HBS | G. Fischer & J. Andriantiana | Madagascar* | 1 |
| *Lichenophylax* | *B. sp. nov.* | FS4002 | HBS | A. Sieder & J. Andriantiana | Madagascar* | 1 |
| *Loxosepalum* | *B. baronii* Ridl. | FS1464 | HBS | G. Fischer, A. Sieder, W. Knirsch & J. Andriantiana | Madagascar* | 1 |
| *Loxosepalum* | *B. leandrianum* H.Perrier | FS5062 | HBS | G. Fischer & J. Andriantiana | Madagascar* | 1 |
|  |  | FS5149 | HBS | G. Fischer & J. Andriantiana | Madagascar* | 1 |
| *Loxosepalum* | *B. lineariligulatum* Schltr. | FS1462 | HBS | G. Fischer, A. Sieder, W. Knirsch & J. Andriantiana | Madagascar* | 1 |
|  |  | FS1581 | HBS | G. Fischer, A. Sieder, W. Knirsch & J. Andriantiana | Madagascar* | 1 |
|  |  | FS1324 | HBS | G. Fischer, A. Sieder, W. Knirsch & J. Andriantiana | Madagascar* | 1 |
|  |  | FS1581 | HBS | G. Fischer, A. Sieder, W. Knirsch & J. Andriantiana | Madagascar* | 1 |
| *Loxosepalum* | *B. nigriflorum* H.Perrier | FS1622 | HBS | G. Fischer, A. Sieder, W. Knirsch & J. Andriantiana | Madagascar* | 1 |
| *Loxosepalum* | *B. sp.* | FS670 | HBS | G. Fischer, A. Sieder & J. Andriantiana | Madagascar* | 1 |
| *Loxosepalum* | *B. sp.* | FS1343 | HBS | G. Fischer, A. Sieder, W. Knirsch & J. Andriantiana | Madagascar* | 1 |
| *Loxosepalum* | *B. sp.* | FS766 | HBS | G. Fischer, A. Sieder & J. Andriantiana | Madagascar* | 1 |
| *Loxosepalum* | *B. sp.* | HBSOR232_2003 | HBS | G. Fischer, A. Sieder & J. Andriantiana | Madagascar* | 1 |
| *Loxosepalum* | *B. sp.* | FS647 | HBS | G. Fischer, A. Sieder & J. Andriantiana | Madagascar* | 1 |
| *Loxosepalum* | *B. sp.* | HBSOR294_2003 | HBS | G. Fischer, A. Sieder & J. Andriantiana | Madagascar* | 1 |
| *Loxosepalum* | *B. sp.* | FS2201 | HBS | G. Fischer & J. Andriantiana | Madagascar* | 1 |
| *Loxosepalum* | *B. sp.* | FS1966 | HBS | G. Fischer & J. Andriantiana | Madagascar* | 1 |
| *Loxosepalum* | *B. sp.* | FS2305 | HBS | G. Fischer & J. Andriantiana | Madagascar* | 1 |
| *Loxosepalum* | *B. sp.* | FS2310 | HBS | G. Fischer & J. Andriantiana | Madagascar* | 1 |
| *Loxosepalum* | *B. sp.* | FS1075 | HBS | G. Fischer, A. Sieder & J. Andriantiana | Madagascar* | 1 |
| *Loxosepalum* | *B. sp.* | FS1044 | HBS | G. Fischer, A. Sieder & J. Andriantiana | Madagascar* | 1 |
| *Loxosepalum* | *B. sp.* | FS1041 | HBS | G. Fischer, A. Sieder & J. Andriantiana | Madagascar* | 1 |
| *Loxosepalum* | *B. sp.* | FS1458 | HBS | G. Fischer, A. Sieder, W. Knirsch & J. Andriantiana | Madagascar* | 1 |
| *Loxosepalum* | *B. sp.* | FS4328 | HBS | G. Fischer & J. Andriantiana | Madagascar* | 1 |
| *Loxosepalum* | *B. sp.* | FS683 | HBS | G. Fischer, A. Sieder & J. Andriantiana | Madagascar* | 1 |
| *Loxosepalum* | *B. sp.* | FS1399 | HBS | G. Fischer, A. Sieder, W. Knirsch & J. Andriantiana | Madagascar* | 1 |
| *Loxosepalum* | *B. vakonae* Hermans | FS1911 | HBS | G. Fischer & J. Andriantiana | Madagascar* | 1 |
| *Loxosepalum* | *B. sp.* | FS2294 | HBS | G. Fischer & J. Andriantiana | Madagascar* | 1 |
| *Loxosepalum* | *B. sp.* | FS2099 | HBS | G. Fischer & J. Andriantiana | Madagascar* | 1 |
| *Loxosepalum* | *B. sp.* | FS2267 | HBS | G. Fischer & J. Andriantiana | Madagascar* | 1 |
| *Loxosepalum* | *B. sp.* | FS1043 | HBS | G. Fischer, A. Sieder & J. Andriantiana | Madagascar* | 1 |
| *Pachychlamys* | *B. sp.* | FS4754 | HBS | A. Sieder, W. Knirsch, Ch. Berg. & M. Pinter | Madagascar* | 1 |
| *Pachychlamys* | *B. molossus* Rchb.f. | FS5531 | HBS | G. Fischer & J. Andriantiana | Madagascar* | 1 |
|  |  | FS5531 | HBS | G. Fischer & J. Andriantiana | Madagascar* | 1 |
| *Pachychlamys* | *B. pachypus* Schltr. | FS1129 | HBS | G. Fischer, A. Sieder, W. Knirsch & J. Andriantiana | Madagascar* | 2 |
| *Pachychlamys* | *B. sandrangatense* Bosser | FS1661 | HBS | G. Fischer & J. Andriantiana | Madagascar* | 1 |
| *Pachychlamys* | *B. sp.* | FS4780 | HBS | A. Sieder, W. Knirsch, Ch. Berg. & M. Pinter | Madagascar* | 1 |
| *Pachychlamys* | *B. vestitum* Bosser | FS5182 | HBS | G. Fischer & J. Andriantiana | Madagascar* | 2 |
|  |  | FS4732 | HBS | A. Sieder, W. Knirsch, Ch. Berg. & M. Pinter | Madagascar* | 1 |
|  |  | FS1347 | HBS | G. Fischer, A. Sieder, W. Knirsch & J. Andriantiana | Madagascar* | 1 |
| *Pachychlamys* | *B. sp.* | FS1965 | HBS | G. Fischer & J. Andriantiana | Madagascar* | 2 |
| *Pachychlamys* | *B. sp.* | FS6085 | HBS | G. Fischer, A. Sieder & J. Andriantiana | Madagascar* | 1 |
| *Pantoblepharon* | *B. onivense* H.Perrier | FS1964 | HBS | G. Fischer & J. Andriantiana | Madagascar* | 1 |
| *Pantoblepharon* | *B. sp.* | FS2932 | HBS | A. Sieder, W. Knirsch & J. Andriantiana | Madagascar* | 1 |
| *Ploiarium* | *B. sp.* | FS685 | HBS | G. Fischer, A. Sieder & J. Andriantiana | Madagascar* | 1 |
| *Ploiarium* | *B. sp.* | FS1984 | HBS | G. Fischer & J. Andriantiana | Madagascar* | 2 |
| *Ploiarium* | *B. sarcorhachis* Schltr. | FS1610 | HBS | G. Fischer, A. Sieder, W. Knirsch & J. Andriantiana | Madagascar* | 1 |
| *Ploiarium* | *B. aggregatum* Bosser | FS5142 | HBS | G. Fischer & J. Andriantiana | Madagascar* | 2 |
|  |  | FS5143 | HBS | G. Fischer & J. Andriantiana | Madagascar* | 2 |
| *Ploiarium* | *B. ankaizinense* (Jum. & H.Perrier) Schltr. | FS1995 | HBS | G. Fischer & J. Andriantiana | Madagascar* | 2 |
| *Ploiarium* | *B. auriflorum* H.Perrier | FS5341 | HBS | G. Fischer & J. Andriantiana | Madagascar* | 2 |
| *Ploiarium* | *B. coccinatum* H.Perrier | FS5537 | HBS | G. Fischer & J. Andriantiana | Madagascar* | 2 |
| *Ploiarium* | *B. conicum* Thouars | FS4757 | HBS | A. Sieder, W. Knirsch, Ch. Berg. & M. Pinter | Madagascar*, Réunion, Mauritius, Comores | 1 |
| *Ploiarium* | *B. coriophorum* Ridl. | FS5082 | HBS | G. Fischer & J. Andriantiana | Madagascar*, Comores | 1 |
|  |  | FS5782 | HBS | G. Fischer, A. Sieder & J. Andriantiana | Madagascar*, Comores | 1 |
| *Ploiarium* | *B. divaricatum* H.Perrier | FS1463 | HBS | G. Fischer, A. Sieder, W. Knirsch & J. Andriantiana | Madagascar* | 1 |
| *Ploiarium* | *B. graciliscapum^1^* H.Perrier | FS945 | HBS | G. Fischer, A. Sieder & J. Andriantiana | Madagascar* | 2 |
|  |  | FS959 | HBV | G. Fischer, A. Sieder & J. Andriantiana | Madagascar* | 1 |
|  |  | FS936 | HBV | G. Fischer, A. Sieder & J. Andriantiana | Madagascar* | 1 |
|  |  | FS964 | HBS | G. Fischer, A. Sieder & J. Andriantiana | Madagascar* | 1 |
|  |  | FS1577 | HBS | G. Fischer, A. Sieder, W. Knirsch & J. Andriantiana | Madagascar* | 1 |
| *Ploiarium* | *B. henrici* Schltr. | FS4725 | HBS | A. Sieder, W. Knirsch, Ch. Berg. & M. Pinter | Madagascar | 1 |
| *Ploiarium* | *B. henrici* Schltr. | FS833 | HBS | G. Fischer, A. Sieder & J. Andriantiana | Madagascar* | 1 |
| *Ploiarium* | *B. hirsutiusculum* H.Perrier | FS4200 | HBS | G. Fischer & J. Andriantiana | Madagascar* | 1 |
| *Ploiarium* | *B. insolitum* Bosser | FS5492 | HBS | G. Fischer & J. Andriantiana | Madagascar* | 1 |
| *Ploiarium* | *B. insolitum* Bosser | FS5492 | HBS | G. Fischer & J. Andriantiana | Madagascar* | 1 |
| *Ploiarium* | *B. masoalanum* Schltr. | FS1383 | HBS | G. Fischer, A. Sieder, W. Knirsch & J. Andriantiana | Madagascar* | 1 |
|  |  | FS2134 | HBS | G. Fischer & J. Andriantiana | Madagascar* | 1 |
|  |  | FS4419 | HBS | G. Fischer & J. Andriantiana | Madagascar* | 1 |
|  |  | FS4222 | HBS | G. Fischer & J. Andriantiana | Madagascar* | 3 |
| *Ploiarium* | *B. nitens* Jum. & H.Perrier | FS5150 | HBS | G. Fischer & J. Andriantiana | Madagascar* | 2 |
|  |  | FS5146 | HBS | G. Fischer & J. Andriantiana | Madagascar* | 1 |
|  |  | FS5203 | HBS | G. Fischer & J. Andriantiana | Madagascar* | 1 |
|  |  | FS2057 | HBS | G. Fischer & J. Andriantiana | Madagascar* | 1 |
|  |  | FS903 | HBS | G. Fischer, A. Sieder & J. Andriantiana | Madagascar* | 1 |
| *Ploiarium* | *B. ormerodianum* Hermans | FS3149 | HBS | A. Sieder, W. Knirsch & J. Andriantiana | Madagascar* | 1 |
| *Ploiarium* | *B. ophiuchus* Ridl. | FS1480 | HBS | G. Fischer, A. Sieder, W. Knirsch & J. Andriantiana | Madagascar* | 1 |
|  |  | FS1480 | HBS | G. Fischer, A. Sieder, W. Knirsch & J. Andriantiana | Madagascar* | 1 |
| *Ploiarium* | *B. peyrotii* Bosser | FS2288 | HBS | G. Fischer & J. Andriantiana | Madagascar* | 1 |
| *Ploiarium* | *B. protectum* H.Perrier | FS4207 | HBS | G. Fischer & J. Andriantiana | Madagascar* | 1 |
| *Ploiarium* | *B. quadrialatum* H.Perrier | FS609 | HBS | - | Madagascar* | 1 |
|  |  | FS609 | HBS | - | Madagascar* | 1 |
| *Ploiarium* | *B. rubiginosum* Schltr. | FS5786 | HBS | G. Fischer, A. Sieder & J. Andriantiana | Madagascar* | 1 |
|  |  | FS800 | HBS | G. Fischer, A. Sieder & J. Andriantiana | Madagascar* | 1 |
| *Ploiarium* | *B. sarcorhachis* Schltr. | FS2046 | HBS | G. Fischer & J. Andriantiana | Madagascar* | 1 |
|  |  | FS1517 | HBS | G. Fischer, A. Sieder, W. Knirsch & J. Andriantiana | Madagascar* | 1 |
| *Ploiarium* | *B. sp.* | FS1983 | HBS | G. Fischer & J. Andriantiana | Madagascar* | 1 |
| *Ploiarium* | *B. sp.* | FS5532 | HBS | G. Fischer & J. Andriantiana | Madagascar* | 1 |
| *Ploiarium* | *B. sp.* | FS3906 | HBS | - | Madagascar* | 1 |
| *Ploiarium* | *B. sp.* | FS5063 | HBS | G. Fischer & J. Andriantiana | Madagascar* | 1 |
| *Ploiarium* | *B. sp.* | FS3914 | HBS | - | Madagascar* | 1 |
| *Ploiarium* | *B. sp.* | FS870 | HBS | G. Fischer, A. Sieder & J. Andriantiana | Madagascar* | 1 |
| *Ploiarium* | *B. sp.* | FS3006 | HBS | A. Sieder, W. Knirsch & J. Andriantiana | Madagascar* | 1 |
| *Ploiarium* | *B. sp.* | FS2347 | HBS | Joseph | Madagascar* | 1 |
| *Ploiarium* | *B. sp.* | FS2092 | HBS | G. Fischer & J. Andriantiana | Madagascar* | 1 |
| *Ploiarium* | *B. sp.* | FS4075 | HBS | A. Sieder & J. Andriantiana | Madagascar* | 1 |
| *Ploiarium* | *B. sp.* | FS2171 | HBS | Joseph | Madagascar* | 1 |
| *Ploiarium* | *B. sp.* | FS2893 | HBS | G. Fischer & J. Andriantiana | Madagascar* | 1 |
| *Ploiarium* | *B. sp.* | FS3887 | HBS | W. Knirsch & J. Adriantiana | Madagascar* | 1 |
| *Ploiarium* | *B. sp.* | FS3147 | HBS | A. Sieder, W. Knirsch & J. Andriantiana | Madagascar* | 1 |
| *Ploiarium* | *B. sp.* | FS992 | HBS | G. Fischer, A. Sieder & J. Andriantiana | Madagascar* | 1 |
| *Ploiarium* | *B. sp.* | FS944 | HBS | G. Fischer, A. Sieder & J. Andriantiana | Madagascar* | 1 |
| *Ploiarium* | *B. sp.* | FS5160 | HBS | G. Fischer & J. Andriantiana | Madagascar* | 2 |
| *Ploiarium* | *B. sp.* | FS5914 | HBS | G. Fischer, A. Sieder & J. Andriantiana | Madagascar* | 1 |
| *Ploiarium* | *B. sp.* | FS2958 | HBS | A. Sieder, W. Knirsch & J. Andriantiana | Madagascar* | 1 |
| *Ploiarium* | *B. sp.* | FS1926 | HBS | G. Fischer & J. Andriantiana | Madagascar* | 1 |
| *Ploiarium* | *B. sp.* | AB192 | HBS | G. Fischer & J. Andriantiana | Madagascar* | 1 |
| *Ploiarium* | *B. sp.* | FS5145 | HBS | G. Fischer & J. Andriantiana | Madagascar* | 2 |
| *Ploiarium* | *B. sp.* | FS1946 | HBS | G. Fischer & J. Andriantiana | Madagascar* | 1 |
| *Ploiarium* | *B. sp.* | FS906 | HBS | G. Fischer, A. Sieder & J. Andriantiana | Madagascar* | 1 |
| *Ploiarium* | *B. sp.* | FS5145 | HBS | G. Fischer & J. Andriantiana | Madagascar* | 1 |
| *Ploiarium* | *B. sp.* | FS5046 | HBS | G. Fischer & J. Andriantiana | Madagascar* | 2 |
| *Ploiarium* | *B. sp.* | FS4739 | HBS | A. Sieder, W. Knirsch, Ch. Berg. & M. Pinter | Madagascar* | 1 |
| *Ploiarium* | *B. sp.* | FS2668 | HBS | G. Fischer & J. Andriantiana | Madagascar* | 2 |
| *Ploiarium* | *B. sp.* | FS4232 | HBS | G. Fischer & J. Andriantiana | Madagascar* | 2 |
| *Ploiarium* | *B. sp.* | FS4872 | HBS | A. Sieder, W. Knirsch, Ch. Berg. & M. Pinter | Madagascar* | 1 |
| *Ploiarium* | *B. sp.* | FS1930 | HBS | G. Fischer & J. Andriantiana | Madagascar* | 1 |
| *Ploiarium* | *B. sp.* | FS930 | HBS | G. Fischer, A. Sieder & J. Andriantiana | Madagascar* | 1 |
| *Ploiarium* | *B. sp.* | FS2941 | HBS | A. Sieder, W. Knirsch & J. Andriantiana | Madagascar* | 1 |
| *Ploiarium* | *B. sp.* | FS3006 | HBS | A. Sieder, W. Knirsch & J. Andriantiana | Madagascar* | 1 |
| *Ploiarium* | *B. sp.* | FS4000 | HBS | A. Sieder & J. Andriantiana | Madagascar* | 1 |
| *Ploiarium* | *B. sp.* | FS2392 | HBS | Joseph | Madagascar* | 2 |
| *Ploiarium* | *B. sp.* | FS4420 | HBS | G. Fischer & J. Andriantiana | Madagascar* | 1 |
| *Ploiarium* | *B. sp.* | FS1558 | HBS | G. Fischer, A. Sieder, W. Knirsch & J. Andriantiana | Madagascar* | 2 |
| *Ploiarium* | *B. sp.* | FS4418 | HBS | G. Fischer & J. Andriantiana | Madagascar* | 1 |
| *Ploiarium* | *B. sp.* | FS2527 | HBS | G. Fischer & J. Andriantiana | Madagascar* | 1 |
| *Ploiarium* | *B. subclavatum* Schltr. | FS653 | HBV | G. Fischer, A. Sieder & J. Andriantiana | Madagascar* | 2 |
|  |  | FS4315 | HBS | G. Fischer & J. Andriantiana | Madagascar* | 1 |
| *Ploiarium* | *B. sp.* | FS1573 | HBS | G. Fischer, A. Sieder, W. Knirsch & J. Andriantiana | Madagascar* | 1 |
| *Ploiarium* | *B. sp.* | FS2782 | HBS | G. Fischer & J. Andriantiana | Madagascar* | 1 |
| *Ploiarium* | *B. sp.* | FS3151 | HBS | A. Sieder, W. Knirsch & J. Andriantiana | Madagascar* | 1 |

^1^A different species with the same name (*B. graciliscapum* Schltr.) is distributed in Southeast Asia (Vanuatu, Papua New Guinea, Solomon Islands).
